# Comprehensive Cell Surface Protein Profiling Identifies Specific Markers of Human Naive and Primed Pluripotent States

**DOI:** 10.1016/j.stem.2017.02.014

**Published:** 2017-06-01

**Authors:** Amanda J. Collier, Sarita P. Panula, John Paul Schell, Peter Chovanec, Alvaro Plaza Reyes, Sophie Petropoulos, Anne E. Corcoran, Rachael Walker, Iyadh Douagi, Fredrik Lanner, Peter J. Rugg-Gunn

**Affiliations:** 1 Epigenetics Programme, The Babraham Institute, Cambridge CB22 3AT, UK; 2 Wellcome Trust – Medical Research Council Cambridge Stem Cell Institute, University of Cambridge, Cambridge CB2 1QR, UK; 3 Department of Clinical Science, Intervention, and Technology, Karolinska Institutet, 14186 Stockholm, Sweden; 4 Division of Obstetrics and Gynecology, Karolinska Universitetssjukhuset, 14186 Stockholm, Sweden; 5 Nuclear Dynamics Programme, The Babraham Institute, Cambridge CB22 3AT, UK; 6 Flow Cytometry Core Facility, The Babraham Institute, Cambridge CB22 3AT, UK; 7 Center for Hematology and Regenerative Medicine, Department of Medicine, Karolinska Institute, 14186 Stockholm, Sweden; 8 Centre for Trophoblast Research, University of Cambridge, Cambridge CB2 3EG, UK

**Keywords:** embryonic stem cells, pluripotency, reprogramming, differentiation, blastocyst, cell surface markers, antibody library

## Abstract

Human pluripotent stem cells (PSCs) exist in naive and primed states and provide important models to investigate the earliest stages of human development. Naive cells can be obtained through primed-to-naive resetting, but there are no reliable methods to prospectively isolate unmodified naive cells during this process. Here we report comprehensive profiling of cell surface proteins by flow cytometry in naive and primed human PSCs. Several naive-specific, but not primed-specific, proteins were also expressed by pluripotent cells in the human preimplantation embryo. The upregulation of naive-specific cell surface proteins during primed-to-naive resetting enabled the isolation and characterization of live naive cells and intermediate cell populations. This analysis revealed distinct transcriptional and X chromosome inactivation changes associated with the early and late stages of naive cell formation. Thus, identification of state-specific proteins provides a robust set of molecular markers to define the human PSC state and allows new insights into the molecular events leading to naive cell resetting.

## Highlights

• Flow cytometry profiles cell surface proteins in naive and primed human PSCs
• The human PSC state can be defined using robust state-specific protein markers
• Identified cell surface proteins track the dynamics of naive-primed PSC conversions
• Analyses of early-stage naive cells reveal transcription events during conversion

## Graphical Abstract

**Figure:**
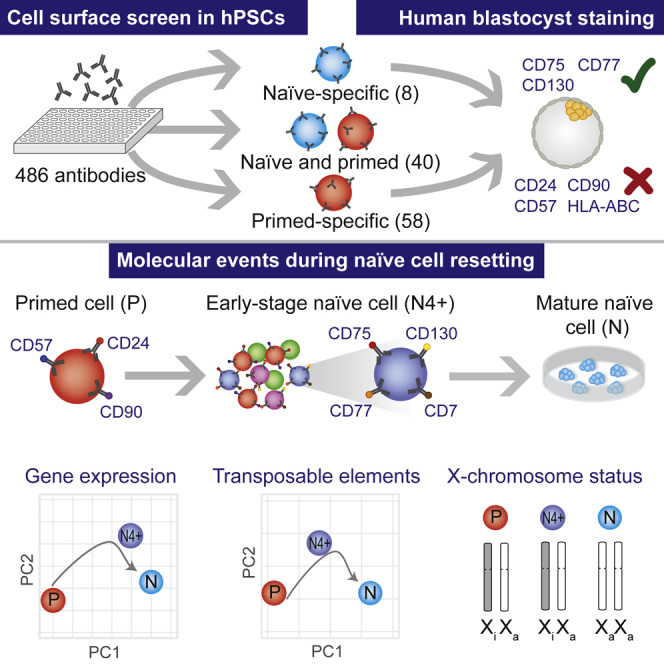

## Teaser

Collier et al. use profiling to identify cell surface proteins that are specific for naive versus primed human pluripotent cells and then use them to isolate and characterize live naive cells arising during primed-to-naive resetting.

## Introduction

Human pluripotent stem cells (PSCs) exist in multiple states of pluripotency that are broadly categorized as naive and primed (Davidson et al., 2015, Weinberger et al., 2016, Wu and Izpisua Belmonte, 2016). Naive and primed PSCs recapitulate several developmental properties of the early- and late-stage human epiblast, respectively, and provide valuable models to investigate the mechanisms that underpin human pluripotency and development (Pera, 2014, Rossant and Tam, 2017). Naive PSCs have been generated by direct derivation from the embryo, through reprogramming of somatic cells or, more commonly, by the conversion of conventional primed PSCs (Chan et al., 2013, Chen et al., 2015, Gafni et al., 2013, Guo et al., 2016, Qin et al., 2016, Takashima et al., 2014, Theunissen et al., 2014, Ware et al., 2014). The current protocols used to convert and maintain naive PSCs vary considerably, resulting in various naive cell types that differ in their gene expression signatures and other properties (Huang et al., 2014). Efforts to define pluripotent states in humans have been challenging, partly because of the variation in naive cell types and partly because detailed molecular characterization of human embryos has only recently been reported (Blakeley et al., 2015, Guo et al., 2014, Okamoto et al., 2011, Petropoulos et al., 2016, Vallot et al., 2017, Yan et al., 2013). By benchmarking properties to the human embryo, a set of standardized molecular criteria to distinguish between naive and primed PSCs has been proposed based on transcriptional and epigenetic profiles (Huang et al., 2014, Theunissen et al., 2016). According to these criteria, naive PSCs maintained in 5 inhibitors, leukemia inhibitory factor (LIF), FGF2 and ActivinA (5i/L/FA) (Theunissen et al., 2014) and titrated 2i/L+Gö6983 (titrated 2 inhibitors, LIF and PKC inhibitor [t2i/L+PKCi]) (Takashima et al., 2014) are classified as being similar to the early-stage human epiblast and are distinct from primed PSCs (Huang et al., 2014, Theunissen et al., 2016). The proposed criteria can interrogate cell populations to infer the PSC state; however, there remains a need to identify standardized markers that are simple and robust and can unambiguously define individual pluripotent cell types within a population.

Monitoring changes in cell state and the emergence of new cell populations are critical for the optimization of protocols and for understanding the mechanisms underpinning the reprogramming process (O’Malley et al., 2013, Polo et al., 2012). Primed-state to naive-state PSC conversion generates a heterogeneous mixture of cells, of which only a small proportion is likely to be naive cells. Current approaches to enrich for a naive cell population include continued passaging under naive culture conditions and the gradual selection of converted cells, manual picking and expansion of individual colonies with characteristic morphology, and the introduction of reporter transgenes into the starting cell type (Gafni et al., 2013, Takashima et al., 2014, Theunissen et al., 2014, Ware et al., 2014). Accurate and transgene-free methods to prospectively identify and isolate naive PSCs from a heterogeneous population are necessary to track the emergence of defined cell types and to capture the cells at earlier stages in their conversion. Two recent studies reported the characterization of individual cell surface markers that can be used to examine naive and primed human PSCs. One study showed that CD24 expression is higher in primed PSCs compared with naive-like cells, and, in combination with the pan-human PSC antigen TRA-1-60, low CD24 levels were used to detect the emergence of a small population of naive-like cells after more than ten passages under naive conditions (Shakiba et al., 2015). A second study reported that the levels of SSEA-4 antigen were low in a subpopulation of naive PSCs that express the highest levels of naive-specific genes (Pastor et al., 2016). Thus, SSEA-4 can be used to purify established naive PSC populations; however, it has not been reported whether the marker can be used to identify emerging naive cells during the conversion process. Currently, no cell surface protein markers that are expressed specifically in naive PSCs have been reported, and, furthermore, it is likely that a combination of cell surface protein markers will be required to unambiguously define PSC states.

Here we describe the results of a large-scale antibody-based screen in naive and primed PSC lines that led to the identification of state-specific cell surface proteins. We validated a cohort of antibodies in multiple naive and primed PSC lines and culture conditions and also found that several naive-specific, but not primed-specific, proteins were expressed in the pluripotent cells of the human preimplantation embryo. We developed an antibody panel targeting multiple cell surface proteins and demonstrated that the panel could distinguish between naive and primed PSCs, track the dynamics of naive-primed interconversion, and isolate emerging naive PSCs from a heterogeneous cell population. The identified cell surface proteins, therefore, provide a standardized and straightforward approach to defining and characterizing state-specific human pluripotent cells.

## Results

### Cell Surface Protein Profiling in Naive and Primed Human PSCs

**Figure 1:**
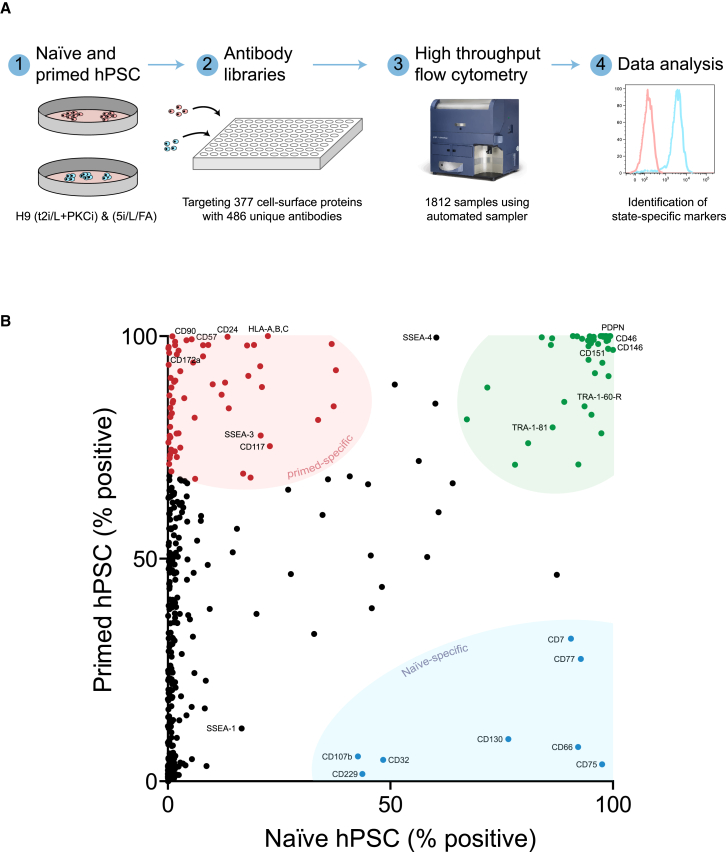
A Resource of Human Naive Cell and Primed PSC Surface Proteins (A) Overview of the experimental design. Human primed (cultured under knockout serum replacement [KSR]/mouse embryonic fibroblast [MEF] and E8/Vitronectin conditions) and naive (cultured under t2i/L+PKCi and 5i/L/FA conditions) H9 PSCs were profiled by multiple antibody libraries that targeted 377 cell surface proteins. Samples were analyzed by high-throughput flow cytometry, and quantification of fluorescence intensity values enabled the identification of state-specific cell surface proteins. See Figure S1 for characterization of the primed and naive PSCs and Figure S2 for additional details regarding the experimental design. (B) Summary of the flow cytometry profiling. Each dot represents a different cell surface protein, and their position along the x and y axes is determined by the percent positive value in naive and primed PSC samples (averaged from one to three independent assays per cell type). Flow cytometry data for naive PSCs cultured under t2i/L+PKCi and 5i/L/FA conditions were combined. Based on their position in the chart, a subset of cell surface proteins have been categorized as naive-specific (blue), primed-specific (red), and common to both naive and primed PSCs (green). See Table S1 for the full dataset. The image of the flow cytometer is provided courtesy of and copyrighted to Becton Dickinson and is reprinted with permission.

Primed human PSCs (H9 line) were converted and maintained in the naive state using two different methods: 5i/L/FA (Theunissen et al., 2014) and t2i/L+PKCi (Takashima et al., 2014), to capture any variation related to resetting and growth conditions (Figure S1). Naive and primed human PSCs were screened against two commercially available cell surface protein antibody panels, which generated data for 486 unique antibodies targeting 377 cell surface proteins (Figure 1A). The percentage of positive cells was determined for each cell surface protein, and values from replicates were averaged (Figure 1B; Table S1).

Providing validation of the experimental approach, our dataset includes several previously reported cell surface markers that are expressed in naive and primed human PSCs, including TRA-1-60 and TRA-1-81 (Chen et al., 2015, Gafni et al., 2013, Pastor et al., 2016, Qin et al., 2016, Shakiba et al., 2015, Ware et al., 2014), SSEA-4 as heterogeneously expressed in naive PSC cultures (Pastor et al., 2016), and CD24 as detected in primed PSCs (Shakiba et al., 2015). Of the many cell surface proteins in our dataset that were newly identified as being expressed in human PSCs, several proteins were detected in both naive and primed PSCs, including PDPN, MCAM (CD146), CD151, and CD46, and will provide a useful set of common markers. The analysis also revealed cell state-specific proteins such as THY1 (CD90), B3GAT1 (CD57), SIRPA (CD172a), and HLA-A,B,C in primed PSCs and CD75, LAMP2 (CD107b), CD7, and LY9 (CD229) in naive-state PSCs (Figure 1B). Notably, the dataset also contained cell state-specific proteins within important functional classes (Table S1). For example, NOTCH receptors were detected only in primed-state PSCs, and the LIF coreceptor (CD130/IL6ST) was detected exclusively in naive-state PSCs, thereby revealing potential differences in signaling pathways between the two pluripotent states. The majority of cell state-specific proteins showed concordant differences in their transcript levels between naive and primed PSCs (21 of 33), although many were discordant between protein and RNA levels, presumably because of post-transcriptional mechanisms (Figure S2D; Table S1). In addition, several cell state-specific markers are glycoproteins and other modified epitopes that cannot be interrogated through transcriptional profiling. Overall, our dataset provides a large-scale resource of cell surface protein expression for naive and primed human PSCs and could be used in future functional studies to interrogate the mechanisms that underpin self-renewal in human pluripotency.

### Validation of Identified Cell Surface Proteins in Multiple PSC Lines and Human Embryos

**Figure 2:**
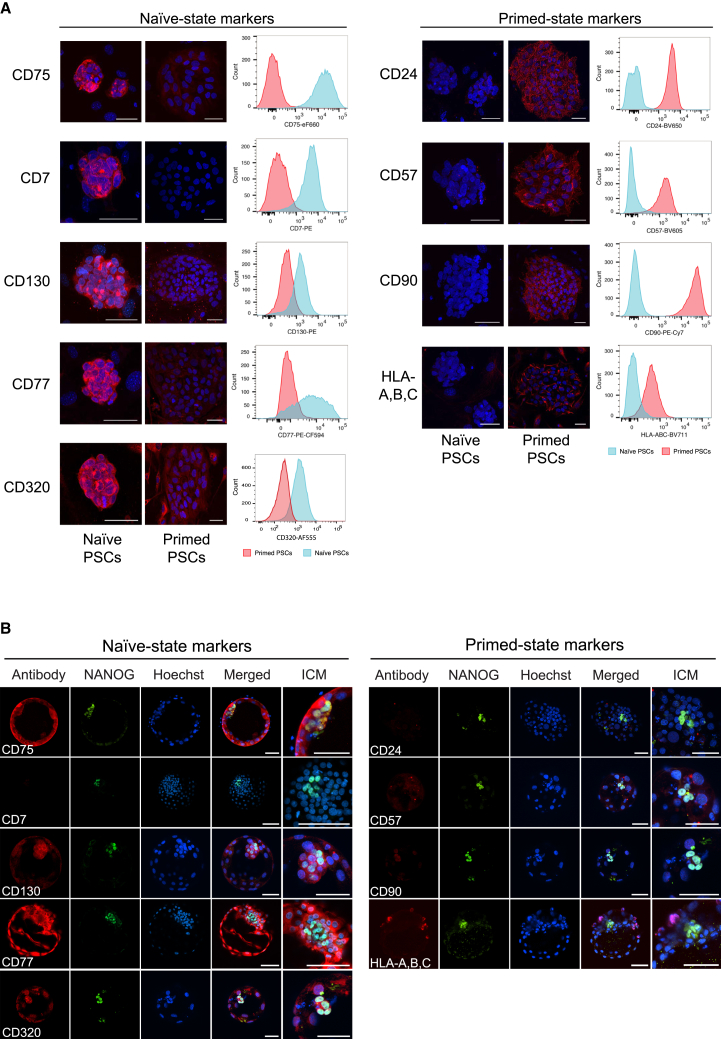
Validation of the Identified Cell Surface Proteins Using Naive and Primed PSCs and Human Blastocysts (A) Immunofluorescent microscopy of primed (KSR/MEF) and naive (5i/L/FA) H9 PSCs for selected cell surface proteins. Histograms of flow cytometry analysis using fluorophore-conjugated antibodies show separation in the fluorescence signal between primed and 5i/L/FA-cultured naive H9 PSCs for all tested cell surface proteins. See Figure S3 for an analysis of t2i/L+PKCi-cultured and RSeT-cultured H9 PSCs. Scale bars, 50 μm. (B) Immunofluorescence microscopy cross-sections of embryonic day 6 human blastocysts labeled with antibodies that detect the identified naive and primed cell surface markers together with NANOG (to reveal the location of epiblast cells) and the DNA stain Hoechst. Scale bars, 50 μm.

We used immunofluorescence microscopy to validate a subset of the newly identified cell surface proteins in primed and 5i/L/FA-cultured naive H9 PSCs. Consistent with their expression profiles obtained from the antibody screen, CD75, CD7, CD77, and CD130 were detected only in naive PSCs and CD24, CD57, CD90, and HLA-A,B,C only in primed PSCs (Figure 2A). In addition, the cell surface protein CD320, which we examined as a potential marker but is not included in the antibody libraries, was expressed in naive PSCs but not in primed PSCs, although some mouse feeder cells showed intracellular staining (Figure 2A). All proteins showed the expected localization at the cell surface of PSCs (Figure 2A). We obtained good separation in fluorescence signal between naive and primed PSCs using flow cytometry analysis of individual markers with fluorescence-conjugated antibodies (Figure 2A). Importantly, we observed similar cell state-specific profiles when comparing primed and t2i/L+PKCi-cultured naive H9 PSCs, demonstrating the robustness of the identified markers (Figure S3A). In contrast, naive-like cells that were generated using RSeT medium displayed a different cell surface marker profile, with a downregulation of two primed-specific proteins (CD24 and CD90) but no upregulation of naive-specific proteins (Figure S3B). Together, these results show that our set of identified cell surface proteins can distinguish between naive cells derived under different conditions and that complete cell resetting under specific culture conditions is required to switch on naive-state cell surface proteins.

The transcriptome of naive PSCs is more similar to cells from human preimplantation embryos than to primed PSCs (Takashima et al., 2014). To investigate whether our identified proteins show a similar stage specificity, we analyzed their expression and localization in embryonic day 6–7 human embryos (Figure 2B). At this time point, all three lineages of the human blastocyst should be established (Petropoulos et al., 2016), and this is also confirmed by the presence of both NANOG-positive epiblast and NANOG-negative primitive endoderm progenitors within the inner cell mass (ICM). Using immunofluorescence microscopy, we could not detect CD7; however, the remaining four naive PSC-specific markers were all expressed in human blastocysts. CD75 and CD77 were detected in the whole embryo, including the ICM, and CD130 and CD320 protein expression was enriched to the ICM, particularly within NANOG-positive epiblast cells (Figure 2B). In contrast, none of the primed PSC-specific proteins CD24, CD57, or CD90 were detected in human preimplantation blastocysts, and HLA-A,B,C was detected only in a few distinct trophectoderm cells (Figure 2B). To validate the expression of the primed PSC-specific markers in postimplantation embryos, we examined a recently published primate transcriptome dataset (Nakamura et al., 2016). This analysis revealed that *CD24*, *CD57*, and *CD90* transcripts are more abundant in postimplantation epiblast cells compared with preimplantation epiblast cells, supporting their classification as primed state markers (Figure S2E). In further agreement with the human blastocyst stainings, *CD130* transcripts were higher in primate preimplantation epiblast cells compared with postimplantation, and *CD7* was not detected at either developmental stage (Figure S2E; CD75 and CD77 are glycoproteins and cannot be assessed by RNA profiling). Overall, the immunofluorescence and transcriptional data confirm that most of the tested naive-specific but few of the primed-specific markers are expressed in preimplantation-stage embryos. Of note is that two of the naive PSC markers (CD75 and CD77) are not localized exclusively in the epiblast but are also present in extraembryonic cells and, by themselves, should not be considered as pluripotent-specific markers in human blastocysts. Nevertheless, taken together, these findings confirm that the identified PSC-specific markers generally reflect developmental stage-specific differences in vivo.

### An Antibody Panel to Distinguish Between Naive and Primed Human PSCs

**Figure 3:**
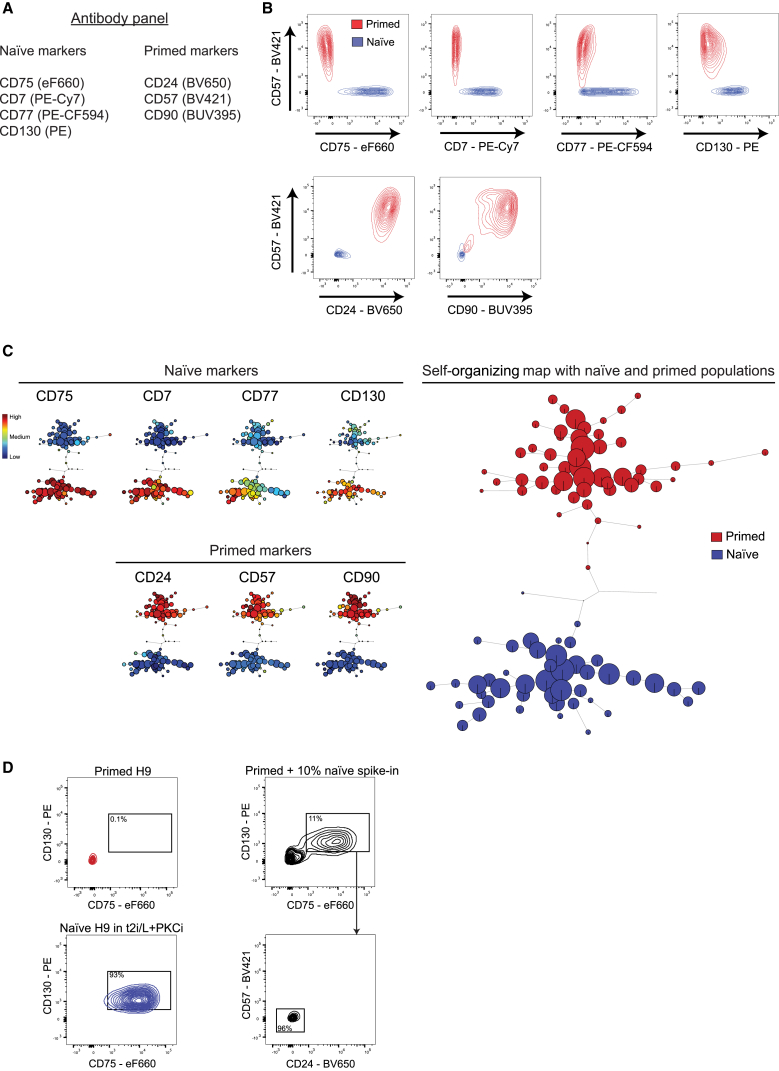
An Antibody Panel to Distinguish between Naive-State and Primed-State Human PSCs (A) A list of antibodies that are combined to form a multiplexed panel. The information in brackets shows the fluorophore conjugation of each antibody. See Table S4 for antibody details and Table S5 for flow cytometer parameters. (B) Flow cytometry contour plots of pairwise antibody combinations. The primed-specific marker CD57 is on the y axes, and different naive-specific (top) and primed-specific (bottom) markers are on the x axes. Primed (red) and t2i/L+PKCi-cultured naive (blue) H9 PSCs are shown for each antibody combination. See Figure S4A for flow cytometry plots that exemplify a typical complete gating scheme for H9 naive PSCs. Note that CD77 shows a greater degree of heterogeneity in naive PSCs compared with the other markers but is still useful when used in combination. (C) FlowSOM visualization of flow cytometry data for all antibodies in the panel. An unsupervised self-organizing map arranges the cells into clusters (represented by circles) according to similarities in their cell surface protein expression profiles (right). Overlaying the identity of the cell type within each cluster reveals a clear separation of naive (blue) and primed (red) populations. The heatmap panels (left) show the expression level of each cell surface protein in the cell clusters. Clusters are arranged in the same position as for the minimal spanning tree of the self-organizing map. See Figures S4B and S4C for analyses of additional ESC and iPSC lines. (D) Flow cytometry contour plots show that the identified panel of state-specific markers can discriminate between primed and naive PSCs when the cells are mixed together. Left: the expression levels of two naive-specific proteins (CD130 and CD75) in primed (top) and naive (bottom) H9 PSCs. Top right: the expression levels of the same proteins in a sample of 90% primed + 10% naive PSCs. Bottom right: CD75^+^/CD130^+^ cells do not express the primed-specific markers CD57 and CD24. Gates were drawn based on unstained, live, human PSCs.

To define a set of cell surface proteins that can discriminate between naive and primed human PSCs, we designed an antibody panel suitable for flow cytometry that multiplexed several of the validated cell state-specific antibodies: CD75, CD7, CD77, CD130, CD24, CD57, and CD90 (Figure 3A). We also included an antibody raised against mouse CD90.2 to detect mouse feeder cells in the samples and kept the GFP spectra available to enable the detection of reporter genes. Flow cytometry analysis showed that combinations of the antibodies can distinguish between naive and primed PSCs, although the range in marker expression within each cell population limits the utility of any individual antibody alone (Figure 3B).

By multiplexing antibodies, we were able to obtain a high-resolution view of the naive and primed PSCs (Figure 3C). We visualized the flow cytometry results using FlowSOM (Van Gassen et al., 2015), which concatenates the data and produces self-organizing maps for clustering and dimensionality reduction. This approach has the advantages of providing a clear overview of the expression level of each marker in all cells and the potential to identify cell subpopulations in an unsupervised manner.

The FlowSOM output for H9 PSCs shows two well separated cell populations that corresponded to naive and primed cells, demonstrating that the antibody panel can discriminate between the two cell states (Figure 3C, right). The individual heatmaps that are projected onto the self-organizing map show the expression levels of each cell surface protein for all cell subpopulations (Figure 3C, left). CD24, CD57, and CD90 expression levels are uniformly high in primed PSCs and low in naive PSCs. Conversely, CD75, CD7, CD77, and CD130 are detected at high to medium levels in naive PSCs and low levels in primed PSCs. We confirmed the antibody panel with additional embryonic stem cell (ESC) and induced pluripotent stem cell (iPSC) lines and also under 5i/L/A and t2i/L+PKCi conditions (Figure S4). Notably, the WIBR3 ESC line carries an OCT4-ΔPE-GFP reporter transgene that is active in naive PSCs (Theunissen et al., 2014), and FlowSOM analysis showed good overlap in the signal between GFP expression and our naive-specific cell surface markers, thereby providing added validation for the antibody panel (Figure S4A).

To more rigorously test the identified protein markers, we investigated whether the antibody panel could discriminate between naive and primed PSCs when the cells were mixed together. We spiked 10% naive PSCs into a sample of primed PSCs, labeled the mixture with our antibody panel, and analyzed the cells by flow cytometry. Gating on CD75^+^/CD130^+^ cells revealed a population corresponding to the naive PSCs, which comprised ∼11% of the sample, suggesting that the majority of spiked-in naive cells were detected (Figure 3D). This population did not express the primed-specific markers CD57 or CD24. Thus, the antibody panel enables the detection of state-specific PSCs in a mixed population and opens up the possibility to prospectively isolate cells during naive-primed PSC transitions.

### Cell Surface Proteins Can Monitor the Dynamics of Naive-Primed PSC Transitions

Naive and primed human PSCs can be interconverted by alteration of culture conditions and reinforced by the short-term expression of key transcription factors such as *NANOG* and *KLF2* (Chan et al., 2013, Gafni et al., 2013, Takashima et al., 2014, Theunissen et al., 2014, Ware et al., 2014). The efficiency of primed-to-naive PSC resetting is variable between protocols and cell lines, but in all cases, substantial cell heterogeneity is generated that could mask the dynamics of cell state changes. Monitoring the changes in cell state and emergence of new cell populations is critical for the optimization of protocols and for understanding the mechanisms underpinning the reprogramming process.

**Figure 4:**
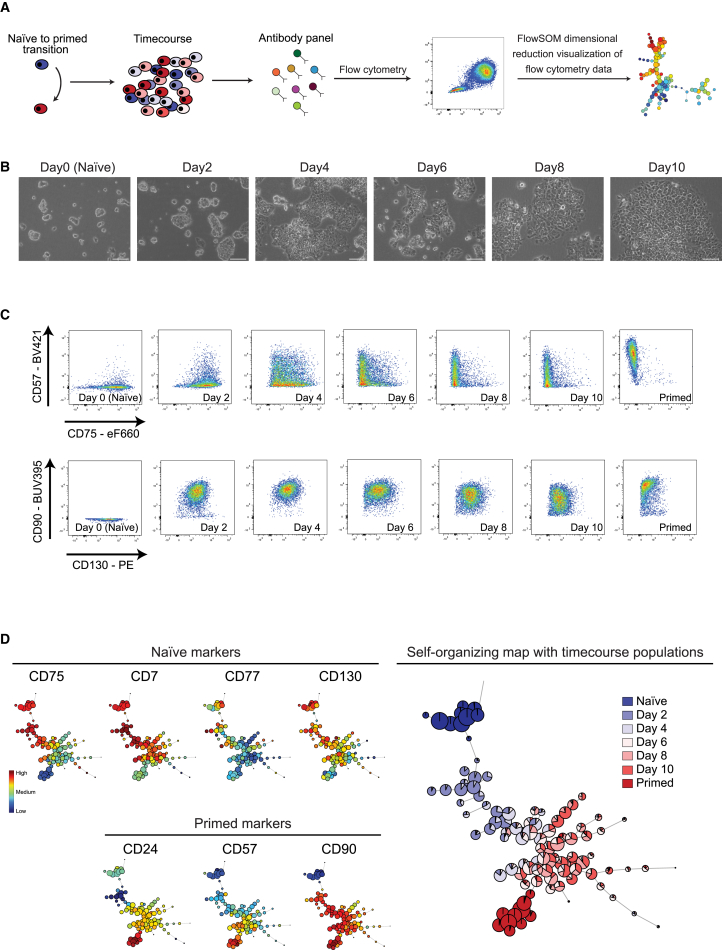
Cell Surface Protein Expression Levels Track the Dynamics of Naive-to-Primed PSC Transition (A) Overview of the experimental design. Shown is a time course experiment of PSCs undergoing a transition from the naive state to the primed state, with flow cytometry analysis every 48 hr. (B) Phase contrast images of H9 PSCs reveal the morphological changes that occur during naive state-to-primed state transition under t2i/L+PKCi conditions. Scale bars, 100 μm. (C) Flow cytometry dotplots of pairwise antibody combinations over the time course. Shown are primed-specific markers on the y axis (CD57, top; CD90, bottom) and naive-specific markers on the x axis (CD75, top; CD130, bottom). (D) FlowSOM visualization of the flow cytometry time course data for H9 PSCs. The minimal spanning tree of the self-organizing map displays an unsupervised clustering of the samples based on their cell surface protein expression levels (right). The results reveal a progressive change in cell surface protein expression during conversion from the naive state to the primed state. The heatmap shows the expression level of each cell surface protein marker in the cell clusters (left).

We first studied the dynamics of cell surface protein expression during naive-to-primed PSC transition (Figures 4A and 4B). Overall, the cell surface markers accurately tracked the cell state change, and, interestingly, each individual protein exhibited different dynamics during the 10-day time course (Figures 4C and 4D). For example, CD90 expression increased sharply within the first 48 hr, whereas upregulation of CD57 was first detected between days 6 and 8. Conversely, CD77 expression was downregulated by day 4, whereas high CD7 and CD130 levels persisted until day 8. Thus, identified cell surface protein markers can be used to track the dynamics of PSCs as they undergo cell state change.

**Figure 5:**
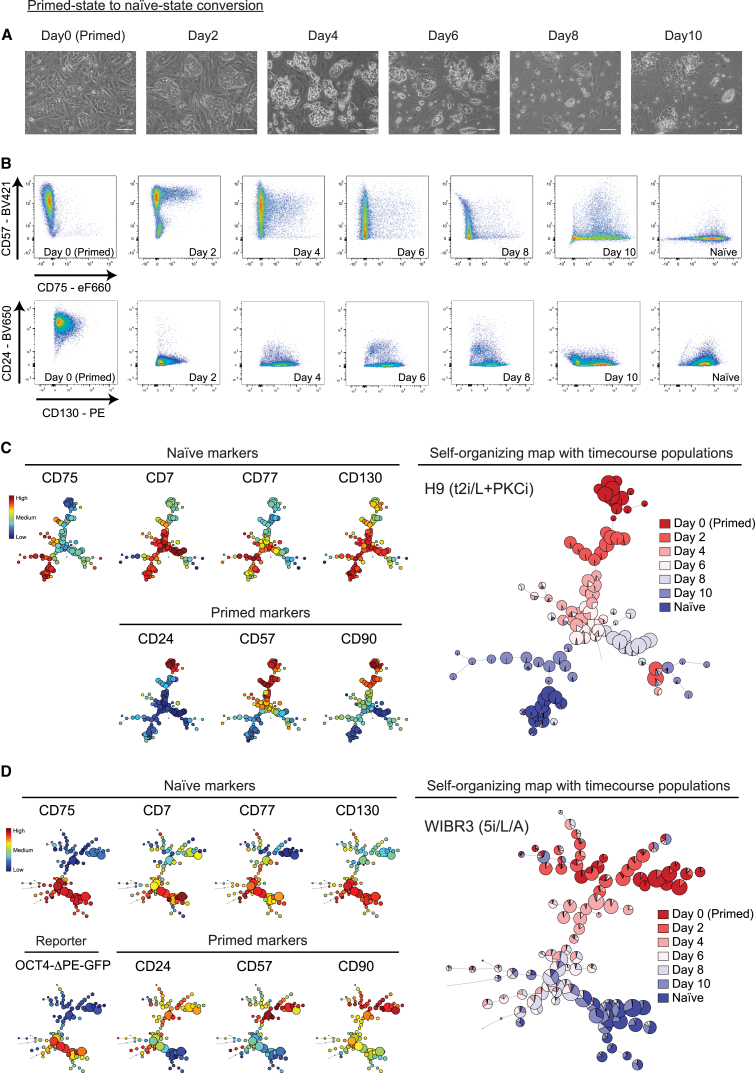
Monitoring the Dynamics of Primed-State to Naive-State PSC Conversion Using Cell Surface Protein Markers (A) Phase contrast images of H9 PSCs reveal the morphological changes that occur during primed-state to naive-state conversion under t2i/L+PKCi conditions. Doxycycline-inducible *NANOG* and *KLF2* transgenes were activated for the first 8 days in t2i/L, and then doxycycline was withdrawn and PKCi was added. Scale bars, 100 μm. (B) Flow cytometry dotplots of pairwise antibody combinations over the time course. Shown are primed-specific markers on the y axis (CD57, top; CD24, bottom) and naive-specific markers on the x axis (CD75, top; CD130, bottom). (C and D) FlowSOM visualization of the flow cytometry time course data for (C) H9 PSCs under t2i/L+PKCi conditions and (D) WIBR3 under 5i/L/A conditions. Note that 5i/L/A conversion is transgene-free and that 5i/L/A was added on day 1. The minimal spanning trees of the self-organizing maps display an unsupervised clustering of the samples based on their cell surface protein expression levels (right). The heatmap shows the expression level of each cell surface protein marker in the cell clusters (left).

We next reset primed H9 PSCs to the naive state using a transient induction of the *NANOG* and *KLF2* transgenes together with t2i/L+PKCi medium (Takashima et al., 2014) and analyzed cell populations by flow cytometry every 48 hr for 10 days (Figure 5A). The expression levels of the primed-specific marker CD57 decreased gradually from high to low over 10 days, with a marked shift occurring as early as day 2 (Figure 5B). In contrast, increased expression of the naive-specific protein CD75 occurred at a late stage during resetting, with expression levels transitioning from low to high between days 8 and 10.

FlowSOM analysis provided additional insights into the dynamics of the primed-to-naive state transitions. Interestingly, the unsupervised self-organizing map staged the cell populations along an axis that largely recapitulated the time course from day 2 to day 10 (Figure 5C). This finding suggests that each time point has a distinct and ordered cell surface protein signature. The FlowSOM heatmaps reveal changes in cell surface protein expression levels during the transition (Figure 5C). For example, CD7 and CD130 are upregulated rapidly upon primed-to-naive transition and reached maximal levels by day 4. CD77 is upregulated more gradually, starting from day 6 onward, and CD75 is upregulated at a late stage during resetting. The primed-specific markers, CD24 and CD90, were downregulated rapidly upon primed-to-naive resetting, and CD57 shifted gradually from high to low over the 10 days. Notably, the greatest spread in the self-organizing map occurred on day 10, reflecting high cellular heterogeneity at this time point. A subset of day 10 cells, however, clustered closely with established naive cells and were likely to be the newly formed naive cells that we characterize in detail in the next sections.

We monitored primed-to-naive resetting using an additional PSC line (WIBR3) and with a transgene-free conversion protocol using 5i/L/A medium (Theunissen et al., 2014). Overall, the cell surface protein markers behaved in a very similar manner (Figure 5D). Interestingly, the efficiency of resetting was noticeably greater using this protocol, and this is reflected by the majority of day 10 cells that are positioned closely to the established naive cells, with a smaller population of day 10 cells that cluster away from naive PSCs. Further validation is provided by the OCT4-ΔPE-GFP reporter signal, which closely overlaps with our naive-state cell surface protein markers (Figure 5D). Taken together, these studies have identified a panel of cell surface protein markers that are able to distinguish between naive and primed human PSCs during differentiation and resetting and thereby provide new ways to investigate the dynamics of cell state transitions.

### Identified Cell Surface Proteins Allow the Prospective Isolation of Early-Stage Naive Cells and the Generation of Naive PSC Lines

**Figure 6:**
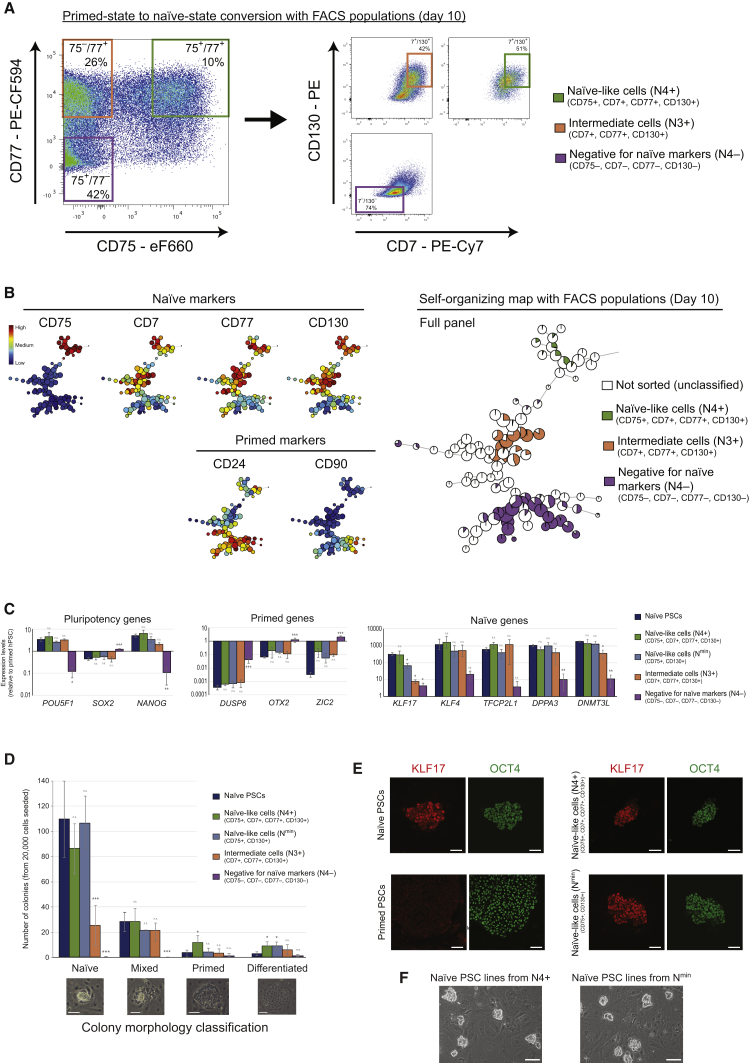
Prospective Isolation of Early-Stage Naive Cells (A) Flow cytometry dotplots of day 10 cells during primed-state to naive-state conversion of H9 PSCs under t2i/L+PKCi conditions. Left: the levels of two naive-specific markers, CD75 and CD77. Based on unstained, live, human day 10 samples, three cell sorting gates have been drawn that correspond to CD75^+^/CD77^+^ (green box), CD75^–^/CD77^+^ (orange box), and CD75^–^/CD77^–^ (purple box) cell populations. Right: the levels of CD7 and CD130 proteins for the same three gated cell populations. Boxed areas indicate the N4^+^ (green), N3^+^ (orange), and N4^–^ (purple) cell populations that were used for subsequent experiments. The percentage of cells within each cell sorting gate relative to all live, human cells is shown. Note that the values do not take into account additional gates; for example, to exclude primed-state markers. See Figure S5C for the N^min^ gating strategy. (B) FlowSOM visualization of the flow cytometry data for day 10 cells during primed to naive conversion. The minimal spanning tree of the self-organizing map displays an unsupervised clustering of the sample based on the cell surface protein expression levels (right). The cells corresponding to each cell sorting population, N4^+^, N3^+^, and N4^–^, are indicated. The heatmap shows the expression level of each cell surface protein marker in the cell clusters (left). See Figure S5A for FlowSOM visualization of WIBR3 PSCs on day 10 of primed state-to-naive state conversion and Figure S5D for FlowSOM visualization of N^min^ cells. (C) qRT-PCR analysis of gene expression levels in the different cell-sorted populations and established naive PSCs. Expression levels are shown on a log scale relative to primed PSCs. Data show the mean ± SD of three or four biological replicates and were compared to established naive PSCs using an ANOVA with Dunnett’s multiple comparisons test (^∗^p < 0.05, ^∗∗^p < 0.005, ^∗∗∗^p < 0.0005). (D) Scoring of colony morphology after transferring the different cell-sorted populations into naive PSC conditions. Colonies were categorized as naive, mixed, primed, and differentiated; examples are shown below. Data show the mean ± SD of three or four biological replicates and were compared to established naive PSCs using an ANOVA with Dunnett’s multiple comparisons test (^∗^p < 0.05, ^∗∗^p < 0.005, ^∗∗∗^p < 0.0005). Scale bars, 100 μm. (E) Immunofluorescence microscopy for KLF17 (a naive-specific protein) and OCT4 (a protein expressed by naive and primed PSCs) reveals that N4^+^ and N^min^ cell-sorted populations can generate KLF17^+^/OCT4^+^ colonies that are similar to established naive PSCs. Scale bars, 100 μm. (F) Phase contrast images showing representative fields of view of N4^+^ and N^min^ cell-sorted populations that have been propagated under t2i/L+PKCi naive PSC conditions for three passages. Scale bars, 100 μm. See Figure S5B for similar results using WIBR3 PSCs under 5i/L/FA conditions.

Primed-to-naive human PSC resetting is an inefficient and variable process and is, therefore, dependent on the accurate detection and isolation of the emerging naive cells. Defining and characterizing partially reprogrammed and intermediate cell states can also provide important insights into the trajectories and mechanisms of cell state changes, as has been demonstrated in iPSC reprogramming (O’Malley et al., 2013, Polo et al., 2012). We investigated whether the cell surface protein markers could prospectively isolate naive cells upon resetting and also capture the cells at an earlier stage in the resetting process than previously possible. Based on our results from the time course experiments described above, we focused on day 10 cells during primed-to-naive resetting. We applied the cell surface antibody panel to the cell population and used cell sorting to isolate cells that expressed all naive-specific protein markers at high levels and were low/off for all primed-specific markers. This population, designated as naive-like cells (N4^+^), represented ∼1% of the total sample (Figures 6A and 6B). For comparison, we also isolated two other cell populations, designated as N3^+^ (CD7^+^, CD77^+^, CD130^+^, and CD75^–^) and N4^–^ (negative for all four naive-specific markers), representing ∼6% and ∼22% of the cell population, respectively (Figures 6A and 6B). Similar cell populations were observed for WIBR3 PSCs using 5i/L/A-mediated conversion, although, of note, the proportion of N4^+^ naive-like cells in the day 10 sample was substantially larger (14%; Figure S5A).

We examined the gene expression profiles of the sorted populations using qRT-PCR. The expression levels of pluripotency factors (*POU5F1*, *SOX2*, and *NANOG*) and naive-specific genes (*KLF17*, *KLF4*, *TFCP2L1*, *DPPA3*, and *DNMT3L*) were similar in N4^+^ cells and established naive PSCs (Figure 6C). As expected, primed-specific genes (*DUSP6*, *OTX2*, and *ZIC2*) were barely detectable in N4^+^ and naive PSCs. Interestingly, the N3^+^ gene expression profile was close to the N4^+^ and naive PSC profiles, with the exception that *KLF17* levels were significantly lower by ∼40-fold (Figure 6C). This finding suggests that N3^+^ cells, which lack CD75 expression, may represent a partially reset cell type, and that *KLF17* is likely to be fully upregulated at the later stages of naive cell formation. In contrast, N4^–^ cells did not display a pluripotent cell gene expression signature, but, instead, their gene expression profile more closely resembled neural-like cells with high levels of *SOX2*, *OTX2*, and *ZIC2* transcripts (Figure 6C). Neural differentiation is consistent with the known response of primed human PSCs to fibroblast growth factor (FGF) inhibitors (Greber et al., 2011), which is one of the components in the resetting medium.

To further characterize the different cell populations, sorted cells were transferred directly into naive PSCs culture conditions, and cell colony morphology was scored after 4 days. The majority of colonies derived from N4^+^ cells were scored as naive-like, with a characteristic compact and domed morphology (344 of 538 colonies, 64%; n = 4; Figure 6D). This proportion is not significantly different from the number of naive-like colonies obtained after plating established naive PSCs under the same conditions (328 of 431, 76%, n = 3). In contrast, significantly fewer naive-like colonies were generated from N3^+^ cells (100 of 220, 45%, n = 4), providing further evidence that these cells are likely to be partially reset. Notably, no naive-like colonies and only four primed-like colonies formed from N4^–^ cells (Figure 6D), which is consistent with their predicted neural fate. Colonies generated from the N4^+^ cells were positive for KLF17 and OCT4 by immunofluorescence microscopy, confirming their status as naive PSCs (Figure 6E). We continued to maintain N4^+^ cells under naive PSC culture conditions for over 20 passages, and the cells generated stable naive PSC lines (Figure 6F). We obtained similar results using the WIBR3 PSC line under 5i/L/FA conditions (Figure S5B).

Multiplexing a large panel of antibodies provides a high-resolution analysis of cell populations but comes with challenges related to ease of use and the availability of suitable flow cytometry equipment. To improve the usability of our approach, we refined the set of antibodies and found that a combination of two naive-specific markers (CD75 and CD130) and two primed-specific markers (CD24 and CD57) could largely recapitulate the full antibody panel. We used this minimal panel to interrogate cells on day 10 of resetting and used cell sorting to isolate cells that were CD75/CD130^high^ and CD24/CD57^low^ (Figures S5C and S5D). This population was designed as N^min^and represented ∼3% of the total sample. Transcriptional analysis of N^min^ cells revealed a gene expression signature that was similar to N4^+^ and naive PSCs (Figure 6C). Furthermore, the cells gave rise to predominantly naive-like colonies in culture (299 of 395, 76%, n = 3) and could form stable naive PSC lines that were KLF17- and OCT4-positive (Figures 6D–6F). Taken together, our results demonstrate that the cell surface markers can identify newly formed naive PSCs from a heterogeneous resetting cell population and that the isolated cells can give rise to established naive PSC lines.

### Distinct Transcriptional and X Chromosome Inactivation Changes Associated with Early and Late Stages of Naive PSC Formation

**Figure 7:**
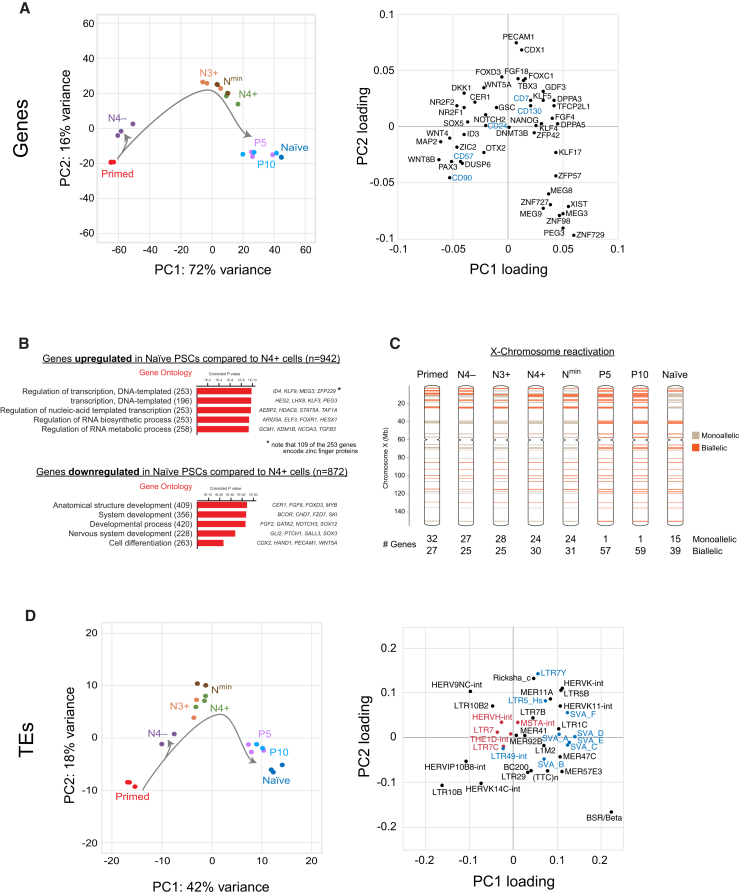
Distinct Molecular Changes during Naive Cell Formation (A) PCA of RNA-sequencing gene expression data from the different cell-sorted populations (left). Right: the contribution of selected genes to the first and second PCs. (B) Top GO terms of genes that were differentially expressed between N4^+^ and established naive PSCs. Numbers of genes are shown; example genes within each GO category are listed (right). Corrected p values were calculated using a modified Fisher’s exact test followed by Bonferroni’s multiple comparisons test. See Table S2 for the full dataset. (C) Schematic of X chromosomes that summarize the results from an allelic analysis of RNA-seq data for the indicated cell types. Informative SNPs within X-linked genes of the H9 PSC line (Vallot et al., 2017) were used to classify expression as monoallelic (brown, <25% from minor allele), biallelic (orange, 25%–75% from minor allele), or not expressed (gray, <10 reads/sample). The number of monoallelic and biallelic genes is shown below. (D) PCA of TE classes from the different cell-sorted populations (left). Right: the contribution of TEs to the first and second PC. Selected TEs are labeled as having a previously defined naive (blue) or primed (red) TE signature (Theunissen et al., 2016).

We used RNA sequencing to assess the transcriptional state of the isolated cell populations and compared them with established naive and primed PSC lines. Clustering by principal-component analysis (PCA) revealed that N4^+^ and N^min^ cells cluster closely to established naive PSCs along the first principal component, which captures 72% of the variation in gene expression (Figure 7A, left). In contrast, N4^–^ cells cluster closer to primed PSCs. The second principal component (capturing 16% of the variation) separates the day 10-isolated populations from the established PSC lines, suggesting that the day 10 samples represent early-stage cell types that have not fully acquired a mature gene expression profile (Figure 7A). To explore this idea further, we profiled isolated N4^+^ cells that were maintained for five passages (P5) and ten passages (P10) in t2i/L+PKCi. PCA showed that these samples aligned closely with established naive PSCs, which demonstrates that the transcriptional program of N4^+^ cells undergoes a final maturation phase over the first few passages under naive culture conditions (Figure 7A).

Examination of genes that contribute to the first principal component reveals the influence of known naive-specific (such as *TFCP2L1*, *DPPA3*, and *KLF4*) and primed-specific (such as *DUSP6*, *OTX2*, and *ZIC2*) genes in segregating the cell clusters (Figure 7A, right). In addition, the influence of genes such as *NR2F2*, *DKK1*, and *SOX5* confirm that N4^–^ cells display a strong neural gene expression signature (Figure 7A). More interestingly, genes that contribute to the second principal component provide new insights into the potential transcriptional differences between early-stage and late-stage naive cells (Figure 7A). For example, genes associated with early-stage N4^+^ cells include *TBX3*, *DPPA3*, *FGF18*, and *FOXC1*, and genes associated with late-stage established naive cells include *XIST*, *MEG3*, and *ZNF729*. Gene ontology (GO) analysis of transcripts that are upregulated in naive PSCs compared with N4^+^ cells revealed a significant enrichment for biological processes related to the regulation of transcription (Figure 7B, top). Strikingly, almost half of the genes within this GO category encode zinc finger proteins (n > 100), suggesting that this class of transcriptional regulator may be associated closely with cell state. Transcripts downregulated in naive PSCs compared with N4^+^ cells are significantly enriched for GO terms related to developmental and differentiation regulators (Figure 7B, bottom). This finding implies that genes potentially involved in lineage priming are robustly silenced during the later stages of naive PSC formation. Taken together, characterization of newly defined cell populations at an early stage in primed-to-naive conversion reveals the transcriptional changes that are associated with naive cell formation and maturation.

Several molecular criteria, including X chromosome status and transposable element (TE) expression, have recently been proposed to provide an accurate approach to distinguish between naive and primed PSCs (Petropoulos et al., 2016, Sahakyan et al., 2017, Theunissen et al., 2016, Vallot et al., 2017). We examined our RNA sequencing (RNA-seq) datasets to determine the allele-specific expression of X-linked genes and then classified informative transcripts as monoallelic or biallelic. This analysis revealed that X chromosome reactivation occurred primarily during the late-stage maturation of naive cells (Figure 7C) and supports the conclusion that X chromosome reactivation is a robust molecular marker of mature naive PSCs. Curiously, this analysis also identified a set of 14 genes on the p arm that were expressed biallelically in the P5 and P10 cells but monoallelically in the established naive PSCs. The reason for this difference is currently unclear but could indicate an erosion of X chromosome activation during long-term maintenance of naive PSCs.

We next investigated the transcription of TEs in the isolated cell populations (Table S3). Clustering of the samples by PCA positioned the N4^+^ cells in between the established primed and naive PSCs, which reinforced our previous result that the day 10 samples represent early-stage cell types that have not fully acquired mature expression profiles (Figure 7D, left). In support of this finding, the P5 and P10 samples clustered closely to the established naive PSCs (Figure 7D). Interestingly, the loadings plot (Figure 7D, right) and clustering analysis (Figure S6) reveal the specific TE families that contribute the most to each sample. In particular, known naive-specific (such as the SVA classes of repeats) and primed-specific (such as LTR7 and HERVH-int) transcripts segregate the first principal component (Figure 7D, right; Theunissen et al., 2016). Moreover, the analysis also identified TE families that may help to characterize early-stage naive cells (such as LTR7Y, LTR5B, and HERV9NC-int) and late-stage naive PSCs (such as MER47C, MER57E3, and BSR/Beta). Taken together, our identified set of cell surface markers and cell sorting strategy have enabled the definition of distinct transcriptional and X chromosome inactivation events associated with naive cell resetting.

## Discussion

We present here the results of a comprehensive antibody screen of cell surface proteins in naive and primed human PSCs. This approach enabled the definition of state-specific cell surface protein signatures that are robust across multiple human PSC lines and culture conditions. The proposed signatures can be applied to interrogate cell populations to infer PSC state. Advantages of this approach over molecular criteria to distinguish between naive and primed PSCs (Theunissen et al., 2016) include the examination of live cells and compatibility with downstream functional assays and the ability to unambiguously categorize individual pluripotent cell types within a population.

Several of the naive-specific but not primed-specific proteins were expressed in preimplantation-stage human embryos, including in pluripotent epiblast cells. This validation provides further reassurance that the naive PSCs resemble human pluripotent cells in vivo, which is in line with previous transcriptional and epigenetic comparisons (Blakeley et al., 2015, Guo et al., 2014, Okamoto et al., 2011, Petropoulos et al., 2016, Theunissen et al., 2016, Vallot et al., 2017, Yan et al., 2013). Nevertheless, differences in protein expression (such as CD7) also raise the possibility that naive PSCs may not entirely recapitulate the properties of human preimplantation epiblast cells, and further research is required to equate PSCs to specific developmental stages. As an initial step, our dataset uncovered several insights that are relevant for the investigation of early-stage human development. To exemplify the application of our dataset to human embryos, we demonstrated that the naive-specific protein CD130 (the LIF co-receptor) is expressed in the human epiblast. Although the role of LIF signaling in mouse development and mouse PSC self-renewal is well established (Ohtsuka et al., 2015, Onishi and Zandstra, 2015), the function of this pathway is poorly understood in human development and PSCs. There are conflicting reports about the expression of LIF signaling components in primed PSCs (Brandenberger et al., 2004, Carpenter et al., 2004, Dahéron et al., 2004, Humphrey et al., 2004), and our work, therefore, provides an impetus for future characterization of this signaling pathway.

Our screening approach enabled us to develop a multiplexed panel of state-specific antibodies that we applied to several critical problems currently encountered during human PSC resetting and differentiation. We first investigated the dynamics of naive-state and primed-state interconversions, which confirmed the utility and specificity of the protein markers and extended our understanding of these cellular processes. In particular, monitoring the changes in cell-surface protein expression allowed the tracking of cell populations and the comparison of different resetting protocols. For example, the proportion of day 10-reset cells with similar protein signatures to established naive PSCs and the timing in the emergence of this cell population were increased under the 5i/L/A conditions compared with t2i/L+PKCi (Takashima et al., 2014, Theunissen et al., 2014). These comparative observations should be useful for the further development of resetting protocols. We also observed differences in the dynamics of each protein marker during a resetting time course. For example, the expression levels of proteins such as CD90 changed rapidly during cell state transitions and are likely to be responsive to cell culture conditions. In contrast, other proteins changed expression more gradually, such as CD130 and CD57, and are therefore more sensitive indicators of cell state. Multiplexing antibodies enabled a high-resolution analysis of cell samples and was able to reveal discrete subpopulations of cells when visualized by dimensionality reduction methods such as FlowSOM (Van Gassen et al., 2015). We suggest that the identified proteins could also be used to study other reprogramming events, such as the conversion of somatic cells to naive iPSCs, or to identify naive PSCs in a screen for naive-promoting factors.

Although the focus of the current study was to identify proteins that can distinguish between naive and primed human PSCs, the availability of an extensive catalog of proteins present on the cell surface of PSCs should also be valuable for the study of human pluripotency and differentiation. In particular, differences in cell surface protein expression raise the possibility that some of the markers may have a role in regulating PSC state. For example, CD75, which was upregulated at a late stage of primed-to-naive resetting, is a cell surface glycoprotein that is catalyzed by sialytransferases (Munro et al., 1992). Sialylation is involved in a variety of cellular functions, such as cell adhesion, signal recognition, and modulation of glycoprotein stability (Pshezhetsky and Ashmarina, 2013, Schauer, 2009). A previous study demonstrated that perturbation of the sialtransferase ST6GAL1 results in less efficient reprogramming of somatic cells and compromised self-renewal of human primed PSCs (Wang et al., 2015). However, sialtransferase activity and function and the role of the glycoprotein CD75 have not been examined in naive PSCs, and this provides one interesting direction for future investigation. Other proteins identified in our screen included several NOTCH receptors that were expressed exclusively in primed PSCs. NOTCH signaling is crucial for many aspects of stem cell regulation, including cell fate decisions and cell proliferation (Perdigoto and Bardin, 2013). It will, therefore, be interesting to investigate whether proteins identified in our screen have a functional role in naive or primed pluripotency. Last, an additional line of future work to enhance our resource could be the application of proteomics, including phosphoproteomics, to the two human PSC-types to obtain a comprehensive overview of protein expression and pathway activity.

Previous studies have relied on transgene expression or the judgement of cell morphology to detect and select naive PSCs (Chan et al., 2013, Gafni et al., 2013, Takashima et al., 2014, Theunissen et al., 2014, Ware et al., 2014). In contrast, our set of cell surface markers can identify and prospectively isolate the emerging naive cells from a heterogeneous population of resetting cells, thereby enabling an unambiguous and straightforward approach to derive naive PSC cultures. This approach also allows the examination of specific cells at a time point much earlier in the resetting process than was previously possible. Our molecular characterization of cells on day 10 of resetting showed that their transcriptome was more similar to naive cells than to primed cells but not identical to established naive PSCs. Importantly, this analysis provided new insights into the temporal sequence of gene expression changes. In particular, we found that the transcription factors *KLF17*, *DPPA5*, and *NANOG* are induced at a relatively late stage of resetting, but other genes, such as *DPPA3* and *TBX3*, are induced earlier. Conversely, the process of X chromosome reactivation and the expression of several genes that define established naive cells such as *MEG3*, *XIST*, and a large set of zinc finger proteins, have not been induced by day 10. Through this approach, we can begin to observe the temporal sequence of molecular events that are triggered during cell resetting, thereby providing a first step toward mapping the route of PSC state transitions. Thus, our work generates an important resource of cell surface proteins in naive and primed PSCs and provides a framework for the future investigation of human pluripotency.

## STAR★Methods

### Key Resources Table

**Table:** 

REAGENT or RESOURCE	SOURCE	IDENTIFIER
**Antibodies**		

Mouse anti-CD130 conjugated to PE (clone AM64)	BD Biosciences	Cat#555757; RRID: AB_396098
Mouse anti-CD130 (clone AM64)	BD Biosciences	Cat#555756; RRID: AB_396097
Mouse anti-CD229 (clone 249936)	R&D Systems	Cat#MAB1898; RRID: AB_2265877
Goat anti-CD229	R&D Systems	Cat#AF1898; RRID: AB_355043
Mouse anti-CD24 conjugated to BV650 (clone ML5)	BD Biosciences	Cat#563720; RRID: AB_2632388
Mouse anti-CD24 conjugated to BUV395 (clone ML5)	BD Biosciences	Cat#563818; RRID: AB_2632389
Mouse anti-CD24 (clone ML5)	BD Biosciences	Cat#555426; RRID: AB_395820
Goat anti-CD320	R&D Systems	Cat#AF1557; RRID: AB_2275689
Mouse anti-CD57 conjugated to BV605 (clone NK-1)	BD Biosciences	Cat#563895; RRID: AB_2632390
Mouse anti-CD57 conjugated to BV421 (clone NK-1)	BD Biosciences	Cat#563896; RRID: AB_2632391
Mouse anti-CD57 (clone NK-1)	BD Biosciences	Cat#555618; RRID: AB_395985
Mouse anti-CD7 conjugated to PE (clone 6B7)	BioLegend	Cat#343106; RRID: AB_1732011
Mouse anti-CD7 conjugated to PE-Cy7 (clone 6B7)	BioLegend	Cat#343114; RRID: AB_2563941
Mouse Anti-CD7 (clone M-T701)	BD Biosciences	Cat#555359; RRID: AB_395762
Mouse anti-CD75 conjugated to eF660 (clone LN1)	eBioscience	Cat#50-0759-42; RRID: AB_2574175
Mouse anti-CD75 (clone LN1)	Abcam	Cat#ab77676; RRID: AB_1566030
Mouse anti-CD77 conjugated to PE-CF594 (clone 5B5)	BD Biosciences	Cat#563631; RRID: AB_2632392
Mouse anti-CD77 (clone 5B5)	BD Biosciences	Cat#551352; RRID: AB_394164
Mouse anti-CD90 conjugated to BUV395 (clone 5E10)	BD Biosciences	Cat#563804; RRID: AB_2632398
Mouse anti-CD90 conjugated to PE-Cy7 (clone 5E10)	BD Biosciences	Cat#561558; RRID: AB_10714644
Mouse anti-CD90 (clone 5E10)	BD Biosciences	Cat#555593; RRID: AB_395967
Rat anti-mouse CD90.2 conjugated to APC-Cy7 (clone 30-H12)	BioLegend	Cat#105328; RRID: AB_10613293
Mouse anti-HLA-ABC conjugated to BV711 (clone G46-2.6)	BD Biosciences	Cat#565333; RRID: AB_2632393
Mouse anti-HLA-ABC (clone G46-2.6)	BD Biosciences	Cat#555551; RRID: AB_395934
Rabbit anti-KLF17	Atlas Antibodies	Cat#HPA024629; RRID: AB_1668927
Goat anti-KLF4	R&D Systems	Cat#AF3158; RRID: AB_2130245
Rabbit anti-NANOG	Abcam	Cat#ab21624; RRID: AB_446437
Rabbit anti-NANOG	ReproCELL	Cat#RCAB0004P-F; RRID: AB_1560380
Mouse anti-POU5F1 (clone C-10)	Santa Cruz	Cat#sc5279; RRID: AB_628051
Mouse anti-SOX2 (clone 245610)	R&D Systems	Cat#MAB2018; RRID: AB_358009
Mouse Anti-SSEA4 (clone MC-813-70)	R&D Systems	Cat#MAB1435; RRID: AB_357704
Goat Anti-TFCP2L1	R&D Systems	Cat#AF5726; RRID: AB_2202564
Mouse anti-β-ACTIN (clone AC-15)	Sigma-Aldrich	Cat#A5441; RRID: AB_476744

**Biological Samples**		

Human embryos at embryonic day 2 or 4	Karolinska University Hospital, Huddinge, Sweden	N/A
Human embryos at embryonic day 2 or 4	Carl von Linné Clinic, Uppsala, Sweden	N/A

**Chemicals, Peptides, and Recombinant Proteins**		

Recombinant human LIF	Millipore	Cat#LIF1050
Recombinant human LIF	WT-MRC Cambridge Stem Cell Institute	N/A
Recombinant Activin A	R&D Systems	Cat#338-AC
Recombinant human bFGF	R&D Systems	Cat#234-FSE-025/CF
PD0325901	Sigma-Aldrich	Cat#PZ0162; CAS: 391210-10-9
PD0325901	WT-MRC Cambridge Stem Cell Institute	CAS: 391210-10-9
WH-4-023	A Chemtek	Cat#0104-002013; CAS: 837422-57-8
IM-12	Sigma-Aldrich	Cat#SML0084; CAS: 1129660-05-1
SB590885	Sigma-Aldrich	Cat#SML0501; CAS: 405554-55-4
Y-27632	Millipore	Cat#688000; CAS: 146986-50-7
CHIR99021	WT-MRC Cambridge Stem Cell Institute	CAS: 252917-06-9
Gö6983	Tocris	Cat#2285; CAS: 133053-19-7

**Critical Commercial Assays**		

BD Lyoplate Human Cell Surface Marker Screening Panel	BD Biosciences	Cat#560747
LEGENDScreen Human Cell Screening Kit	BioLegend	Cat#700001

**Deposited Data**		

RNA-seq data deposited to Gene Expression Omnibus	This paper	GEO: GSE93241
Human reference genome NCBI build 38, GRCh38	Genome Reference Consortium	http://www.ncbi.nlm.nih.gov/projects/genome/assembly/grc/human/
RNA-sequencing data used for calling concordant expression between gene and protein levels (related to Figure S2 and Table S1)	Takashima et al., 2014	ArrayExpress: E-MTAB-2857
Primate transcriptional data from Gene Expression Omnibus (related to Figure S2E)	Nakamura et al., 2016	GEO: GSE74767
Positions of heterozygous SNPs on the X-chromosome of H9 cells (related to Figure 7)	Provided by Celine Vallot; Vallot et al., 2015	N/A

**Experimental Models: Cell Lines**		

WA09/H9 primed PSCs	WiCell	WA09
WA09/H9 NK2 naive and primed PSCs	Laboratory of Austin Smith; Takashima et al., 2014	N/A
WA09/H9 FiPS naive and primed PSCs	Laboratory of Austin Smith; Takashima et al., 2014	N/A
WIBR3 naive and primed PSCs	Laboratory of Rudolph Jaenisch; Theunissen et al., 2014	N/A

**Recombinant DNA**		

piggyBac transposon vector	Zhang et al., 2015	N/A
piggyBac EGFP transposase vector	Zhang et al., 2015	N/A

**Sequence-Based Reagents**		

For primer sequences, please see Table S6		
NEBNext Ultra RNA Library Prep Kit	NEB	E7530
Poly(A) mRNA Magnetic Isolation Module	NEB	E7490

**Software and Algorithms**		

FlowJo V10.1	FlowJo LLC	https://www.flowjo.com/solutions/flowjo
FlowAI V1.2.4	Monaco et al., 2016	https://www.bioconductor.org/packages/release/bioc/html/flowAI.html
FlowSOM V1.2.0	Van Gassen et al., 2015	https://www.bioconductor.org/packages/release/bioc/html/FlowSOM.html
HISAT 2.0.5	Kim et al., 2015	https://ccb.jhu.edu/software/hisat2/index.shtml
Seqmonk v36.0	Babraham Institute, Bioinformatics	http://www.bioinformatics.babraham.ac.uk/projects/seqmonk/
DESeq2	Love et al., 2014	https://bioconductor.org/packages/release/bioc/html/DESeq2.html
ImageJ	ImageJ	https://imagej.nih.gov/ij/
Ensembl Genome Browser v70	Aken et al., 2017	http://www.ensembl.org/index.html
Trim Galore v0.4.2	Babraham Institute, Bioinformatics	http://www.bioinformatics.babraham.ac.uk/projects/trim_galore/
Samtools	Li et al., 2009	http://samtools.sourceforge.net/
SNPsplit v0.3.1	Krueger and Andrews, 2016	https://github.com/FelixKrueger/SNPsplit
AmiGO 2	Carbon et al., 2009	http://amigo.geneontology.org/amigo
GraphPad Prism 7	GraphPad Software	https://www.graphpad.com/

### Contact for Reagent and Resource Sharing

Further information and requests for resources and reagents should be directed to and will be fulfilled by the Lead Contact, Fredrik Lanner (fredrik.lanner@ki.se).

### Experimental Model and Subject Details

#### Cell lines

WA09/H9 primed cells were obtained from WiCell. WA09/H9 NK2 and FiPS naive and primed PSCs were kindly provided by Austin Smith (Takashima et al., 2014) with permission from WiCell. Naive and primed WIBR3 PSCs were kindly provided by Rudolph Jaenisch (Theunissen et al., 2014). All PSCs were cultured in 5% O_2_, 5% CO_2_ at 37°C.

#### Embryos

Human embryos were obtained from the Karolinska University Hospital, Huddinge and from the Carl von Linné Clinic, Uppsala, either frozen at embryonic day (E) 2 or from preimplantation genetic diagnosis testing at E4, with informed consent from donating couple and with ethical approval for these experiments to F.L. from the Regional Ethics Board, Stockholm (2012/1765-31/1). Frozen embryos were thawed with ThawKit Cleave (Vitrolife) into G-1 Plus medium (Vitrolife) covered with Ovoil (Vitrolife) and from E3 embryos were cultured in G-2 Plus medium until E6-7 in 5% O_2_, 5% CO_2_ at 37°C.

### Method Details

#### Cell culture

Primed PSCs were maintained in DMEM/F12, 20% Knockout Serum Replacement, 1% nonessential amino acids, 2mM GlutaMAX, 50 U/ml and 50 μg/ml penicillin-streptomycin (all from ThermoFisher Scientific), 0.1mM β-mercaptoethanol (Millipore), and 4–8ng/ml basic fibroblast growth factor (bFGF; R&D Systems) on irradiated mouse embryonic fibroblasts (MEF) seeded at a density of 1x10^6^ cells per 6-well plate. Cells were passaged by 5min incubation with 200U/ml Collagenase type IV (ThermoFisher Scientific). For feeder-free culture, primed PSCs were transferred onto Vitronectin-coated plates (0.5μg/cm^2^; ThermoFisher Scientific) in complete TeSR-E8 or mTeSR1 medium (StemCell Technologies). Cells were passaged by 6min incubation at room temperature with Gentle Cell Dissociation Reagent (StemCell Technologies).

Naive PSCs cultured in 5i/L/FA and 5i/L/A conditions were maintained as previously described (Theunissen et al., 2014, Theunissen et al., 2016) in 1:1 mixture of DMEM/F12 and Neurobasal, 1x N2-supplement, 1x B27-supplement, 1% nonessential amino acids, 2mM GlutaMAX, 50 U/ml and 50 μg/ml penicillin-streptomycin (all from ThermoFisher Scientific), 0.1mM β-mercaptoethanol (Millipore), 50 μg/ml bovine serum albumin (Sigma), 0.5% Knockout Serum Replacement, 20ng/ml recombinant human LIF (Millipore), 20ng/ml Activin A (R&D Systems), 8ng/ml bFGF (omitted in 5i/L/A conditions), 1 μM PD0325901 (Sigma), 1 μM IM-12 (Sigma), 1 μM WH-4-023 (A Chemtek), 0.5 μM SB590885 (Sigma), 10 μM Y-27632 (Millipore) on a MEF-layer seeded at a density of 2x10^6^ cells per 6-well plate. Cells were passaged with 5min incubation with Accutase (ThermoFisher Scientific). Naive PSCs cultured in t2i/L+PKCi conditions were maintained as previously described (Takashima et al., 2014) in a 1:1 mixture of DMEM/F12 and Neurobasal, 0.5x N2-supplement, 0.5x B27-supplement, 1x nonessential amino acids, 2mM L-Glutamine, 1x Penicillin/Streptomycin (all from ThermoFisher Scientific), 0.1mM β-mercaptoethanol (Sigma-Aldrich), 1 μM PD0325901, 1 μM CHIR99021, 20ng/ml human LIF (all from WT-MRC Cambridge Stem Cell Institute) and 2 μM Gö6983 (PKCi; Tocris) on a MEF-layer seeded at a density of 2x10^6^ cells per 6-well plate. For feeder-free culture, t2i/L+PKCi naive PSCs were cultured on Matrigel–coated plates (Corning).

For conversion to 5i/L/FA and 5i/L/A naive PSCs, primed PSCs were dissociated into single cells with Accutase and 2x10^5^ cells per 6-well were plated in primed PSC media with 10 μM Y-27632 onto MEF seeded at a density of 2x10^6^ cells per 6-well plate. The following day, media was changed to 5i/L/FA or 5i/L/A media. Dome-shaped naive colonies could be seen as early as four days after plating, and cells were passaged with Accutase on day 6 for 5i/L/A cells, and on day 10 for 5i/L/FA cells.

For conversion to t2i/L+PKCi naive PSCs, primed H9 NK2 PSCs were dissociated into single cells with Accutase and 2x10^5^ cells per 6-well were plated in primed PSC media with 10 μM Y-27632 onto MEF seeded at a density of 2x10^6^ cells per 6-well plate. The following day (day 1), media was changed to primed PSC media with 1 μg/ml doxycycline (DOX). On Day 2, media was changed for 2i/L+DOX media and replaced daily until day 8 where media was changed to t2i/L+PKCi. Cells were passaged using Accutase on day 3 (1:5 ratio) and day 7 (1:3 ratio).

Conversion of primed H9 NK2 PSCs to naive-like PSCs in RSeT media was achieved following the manufacturer’s manual (StemCell Technologies). This media formation is based on a bFGF– and TGFβ–free version of NHSM (Gafni et al., 2013). Cells were assayed after 4 passages in RSeT conditions.

For transition to primed PSCs, 5i/L/FA-cultured naive PSCs were dissociated into single cells with Accutase and 2x10^5^ cells per 6-well were plated in 5i/L/FA naive PSC media onto MEF seeded at a density of 1x10^6^ cells per 6-well plate. The following day, media was changed to primed PSC media. Flat primed PSC colonies could be seen as early as four days after plating and cells were passaged around 10 days after plating using Collagenase type IV. For transition to primed PSCs, t2i/L+PKCi-cultured naive PSCs maintained in feeder-free conditions were dissociated into single cells with Accutase, and 2x10^5^ cells per 6-well were plated in t2i/L+PKCi naive PSC media onto Matrigel-coated 6-well plates. Two days later (day 0), media was changed to complete mTeSR1 medium. Cells were passaged on day 4 using Accutase for the first passage, and Collagenase type IV for subsequent passages.

#### GFP transfection

For establishing stable GFP expressing cells, primed H9 PSCs were cultured on plates coated with human PSC-qualified Matrigel in mTeSR1 medium. Cells were co-transfected in a 6-well format with 2.5 μg *piggyBac* transposon and 2.5 μg *EGFP transposase* vectors (Zhang et al., 2015) using 10 μL Lipofectamine LTX (ThermoFisher Scientific) and 5 μL PLUS reagent (ThermoFisher Scientific) according to the manufacturer’s instructions. 48h after transfection, selection with 1 μg/ml puromycin (ThermoFisher Scientific) was started for a 6-day period. Transfected cells were transferred back onto MEFs and maintained in primed PSC culture conditions or converted into 5i/L/FA naive PSCs.

#### Cell-surface marker screening

Antibody screening was performed on H9 PSCs cultured on feeders and feeder-free, with naive-state PSCs cultured in two different conditions: 5i/L/FA and t2i/L+PKCi.

For 5i/L/FA cells, GFP-expressing primed and naive PSCs were dissociated into single cells with Accutase and passed through 40 μm cell strainer (BD Biosciences). For barcoding, primed PSCs were washed with PBS and the cell concentration was adjusted to 1x10^6^ cells/ml in prewarmed (37°C) PBS. CellTrace Violet (ThermoFisher Scientific) was added to the cell suspension to obtain 5 μM solution and cells were incubated for 20min in a 37°C water bath, protected from light. Cells were washed 3x with buffer (2% FBS in PBS) and centrifugation at 300xg for 5min. 5i/L/FA-cultured naive PSCs and barcoded primed PSCs were combined at a 1:1 ratio (3.5x10^6^ cells each in 28ml buffer). 100 μL of cell mixture was aliquoted into V-bottom 96-well plates (BD Falcon) and 20 μL of reconstituted antibodies were added to wells from Human Cell Surface Marker Lyoplates (BD Biosciences 560747) and incubated for 20min on ice. Cells were washed 2x with buffer and centrifuged at 300xg for 5min. Secondary antibodies conjugated to Alexa Fluor 647 and diluted 1:200 with buffer were applied to the cells and incubated for 20min on ice. Cells were washed 2x with buffer and centrifuged at 300xg for 5min. Cells were resuspended in buffer with 1:200 7-AAD (BD Biosciences) and analyzed with BD LSRFortessa cell analyzer (BD Biosciences).

For t2i/L+PKCi cells, primed and naive PSCs were maintained in feeder-free conditions and dissociated into single cells with Accutase. Cells were washed with either primed or naive PSC media, collected by centrifugation at 300xg 5min and resuspended in Cell Staining Buffer (BioLegend). Cells were passed through a 30 μm cell strainer (Sysmex) and the cell concentration was adjusted to ∼1.8x10^6^ cells/ml with a total volume of 30ml. 75 μL containing 1.5x10^5^ cells was aliquoted into each well of LEGENDScreen Human Cell Screening 96-well plates (BioLegend 700001), containing 25 μL reconstituted PE-conjugated antibody. Plates were incubated for 30 min at 4°C protected from light. Plates were washed 2x with 200 μL Cell Staining Buffer per well, before final resuspension in 160 μL Cell Staining Buffer containing 1 μg/ml DAPI. Cells were analyzed using a BD LSRFortessa cell analyzer (BD Biosciences). The antibody screen was performed in biological duplicate for naive and primed PSCs.

#### Flow cytometry

Primed and naive PSCs were dissociated into single cells with Accutase, washed and passed through 30-40 μm cell strainers. Conjugated antibodies were mixed with 50 μL Brilliant stain buffer (BD Biosciences) and applied to 50-100 μL of cells (2-5x10^5^ cells per reaction). Cells were incubated for 30min at 4°C in the dark and washed 2x with buffer (2% FBS in PBS) and centrifuged at 300xg for 5min. Cells were resuspended in buffer with 7-AAD (BD Biosciences) or DAPI (Sigma) and analyzed with a BD LSRFortessa cell analyzer (BD Biosciences) or a BD FACSAria Fusion for cell sorting. Single-stained cells or OneComp eBeads (eBioscience) were used for compensation calculations. Unstained cells, GFP-expressing cells with 7-AAD, and Fluorescence Minus One (FMO) controls were used in cytometer and gating set up. Data was analyzed using FlowJo V10.1 software (FlowJo, LLC). Antibody details can be found in Table S4.

Cell sorting was performed on day 10 transitioning cells. We noticed that the PKCi Gö6983 produces a strong autofluorescent signal, therefore, unstained H9 NK2 naive-state PSCs were used to setup the flow cytometer when sorting H9 NK2 transitioning cells in order to provide a comparable fluorescence signal. Cells for continued culture were sorted directly into t2i/L+PKCi plus Y-27632. Cells were cultured in the presence of Y-27632 for 12-24hr, and then media was replaced with t2i/L+PKCi. As there is no PKCi-mediated autofluorescent signal in WIBR3 cultures, unstained WIBR3 primed-state PSCs were used to setup the flow cytometer when sorting WIBR3 transitioning cells. Day 10 WIBR3 cells for continued culture were initially sorted into 5i/L/A media. Cell viability was fine, but we noticed that cells failed to expand in number upon subsequent passages. We therefore sorted WIBR3 cells into 5i/L/FA media, which helped but failed to completely resolve this issue. In the end, we found that performing two-third media changes substantially enhanced cell number expansion. Based on this, we recommend that WIBR3 day 10 cells are sorted directly into 5i/L/FA media, and after two passages, switch the media to 5i/L/A for continued culture using two-third media changes.

Table S5 shows information related to the setup of the flow cytometers including details of lasers, filters and fluorochromes.

#### FlowSOM analysis

FCS files were run through FlowAI V1.2.4, a quality control package that filters events affected by technical variation, such as abrupt flow rate fluctuations (Monaco et al., 2016). Gating was performed in FlowJo V.10.1 (FlowJo, LLC). Subsequent analysis was performed using FlowSOM (V1.2.0), an R bioconductor package that uses self-organizing maps for dimensional reduction visualization of flow cytometry data (Van Gassen et al., 2015). All data was scaled and logicle transformed on import. Cells were assigned to a Self-Organizing Map (SOM) with a 10x10 grid, which groups similar cells into 100 clusters. To visualize similar clusters a minimal spanning tree (MST) was constructed and cell counts were log scaled.

For the time course experiments, live-human-gated cell populations from each day were exported. To account for cell number variation, an equal number of cells were exported for each time point. The same analysis was performed for the comparison of naive and primed PSCs. For the analysis of cell sorted samples, the N4+, N3+ and N4- populations were additionally exported and visualized on the minimal spanning tree (MST) constructed using all live human cells.

#### Immunofluorescent microscopy

Cells were fixed with 4% formaldehyde (Polysciences) for 15min and permeabilized with 0.3% Triton X-100 (Sigma) in PBS for 10min. Cells were blocked with 5% donkey serum (Abcam), 1% BSA, and 0.1% Tween-20 (Sigma) in PBS for 1h. Primary antibodies were applied for overnight at 4°C. Cells were washed with 0.1% BSA, and 0.1% Tween-20 in PBS and secondary antibodies were applied for 1h. Primary and secondary antibodies were diluted in 1% donkey serum, 0.1% BSA, and 0.1% Tween-20 in PBS. DNA was counterstained with 1 μg/ml Hoechst 33342 (ThermoFisher Scientific) for 15min.

Human embryos were fixed with 4% formaldehyde for 15min and permeabilized with 0.3% Triton X-100 in PBS for 10min. Blocking was performed overnight at 4°C in 4% FBS (ThermoFisher Scientific) and 0.1% Tween 20 in PBS. Embryos were incubated with primary antibodies overnight at 4°C in blocking buffer, followed by 3x 5min washes in blocking buffer. Embryos were incubated with secondary antibodies overnight at 4°C in blocking buffer, followed by 3x 5min washes in blocking buffer, and 20min incubation with 1 μg/ml Hoechst 33342. Embryos were mounted in blocking buffer between two coverslips using Secure-Seal adhesive spacers (ThermoFisher Scientific).

Images were collected on a Zeiss LSM710-NLO point scanning confocal microscope with a 20x water immersion objective, and on a NIKON A1-R confocal microscope with a 40x oil objective. Z stack images were processed with ImageJ (https://imagej.nih.gov/ij/). Antibody details can be found in Table S4.

#### Western blotting

Whole cell lysates were extracted in RIPA buffer (Sigma) with 1x cOmplete Mini EDTA-free Protease Inhibitor Cocktail (Roche). Proteins were separated by electrophoresis using 12% SDS-polyacrylamide gels and transferred to 0.45 μM PVDF membranes (Amersham Hybond). Membranes were blocked for > 3h in TBS-T 5% milk and hybridized to primary antibody overnight at 4°C. Membranes were washed 3x for 10min in TBS-Tween 5% milk at room temperature then incubated for 1h at room temperature with secondary antibodies HRP-conjugated goat-anti-mouse, goat-anti-rabbit (1:5,000, Bio-Rad) or donkey-anti-goat (1:2,500, Jackson ImmunoResearch) immunoglobulins. Detection was performed using Clarity Western ECL reagent (Bio-Rad). Primary antibodies: TFCP2L1 (1:500, R&D Systems AF5726); KLF17 (1:200, Atlas Antibodies HPA024629); POU5F1 (1:500, Santa Cruz sc5279); β-ACTIN (1:1000, Sigma A5441).

#### qPCR

For data in Figure S1, RNA was extracted using the RNeasy Mini Kit (QIAGEN), and 1 μg RNA was reverse transcribed using SuperScript III (ThermoFisher Scientific) followed by quantitative PCR with Taqman universal master mix and Taqman assays (ThermoFisher Scientific) using the StepOnePlus Real-Time PCR System (ThermoFisher Scientific). For data in Figure 6, cells isolated by cells sorting went directly into TRIzol LS Reagent (ThermoFisher Scientific) and total RNA was extracted. RNA (1 μg) was reverse transcribed using the QuantiTect Reverse Transcription Kit (QIAGEN), and quantitative PCR was performed using JumpStart Sybr Green (Sigma). RNA from three or four biological replicates were used for each condition. For primer information, see Table S6.

#### RNA-sequencing

For RNA-sequencing, indexed libraries were constructed from ∼500ng total RNA using the NEBNext Ultra RNA Library Prep Kit for Illumina with the Poly(A) mRNA Magnetic Isolation Module (NEB). Library fragment size and concentration was determined using an Agilent Bioanalyzer 2100 and KAPA Library Quantification Kit (KAPA Biosystems). Samples were sequenced on an Illumina NextSeq 500 instrument as 150bp single-end libraries at the Babraham Institute Sequencing Facility.

RNA-sequencing reads were trimmed using trim galore v0.4.2 (http://www.bioinformatics.babraham.ac.uk/projects/trim_galore/) using default parameters to remove the standard Illumina adaptor sequence. Reads were mapped to the human GRCh38 genome assembly using HISAT 2.0.5 (Kim et al., 2015) guided by the gene models from the Ensembl v70 release (Aken et al., 2017). Samtools (Li et al., 2009) was used to convert to BAM files that were imported to Seqmonk v36.0 (http://www.bioinformatics.babraham.ac.uk/projects/seqmonk/). Raw read counts per transcript were calculated using the RNA-sequencing quantitation pipeline on the Ensembl v70 gene set using directional counts. Biological replicates were merged and differentially expressed genes were determined using DESeq2 (Love et al., 2014). Regularised log transformation was applied prior to visualization to correct for library size and variance among counts. PCA was performed using the top 1000 most variable genes across experimental condition. The first and second principal components were plotted. Gene Ontology (GO) analysis for genes differentially expressed between N4+ and established naive PSCs was performed using AmiGO 2 (Carbon et al., 2009) with default settings.

#### Transposable element analysis

To analyze the expression of transposable elements, probes were generated in SeqMonk over the locations of hg38 repeats and then filtered to remove those which were within 2kb of a gene. Raw counts for all of the reads that overlapped with the final probe set were exported and collated to generate counts for each class of repeat. Reads were globally normalized per million reads. Samples containing > 3% reads outside of genes were discarded due to potential DNA contamination that could mask the quantification of transposable elements. PCA was performed using the count data for repeat classes containing a minimum of 20 total reads across the samples. The first and second principal components were plotted using the top 1000 most variable transposable elements across experimental condition.

#### Quantification of X-linked genes

To analyze allele-specific expression of X-linked genes, an N-masked genome was generated using the positions of heterozygous SNPs on the X chromosome of H9 cells (coordinates kindly provided by Celine Vallot (Vallot et al., 2015). RNA-sequencing reads were trimmed using trim galore v0.4.2 and aligned to the N-masked genome using HISAT2 (default settings but without soft-clipping). The mapped data was sorted into allele-specific reads using SNPsplit (v0.3.1, default parameters, single-end) (Krueger and Andrews, 2016). Genome1/genome2 reads, which corresponded to reads carrying either of the two SNPs, were imported into SeqMonk. Probes were designed over informative SNP annotations (provided by Celine Vallot) and quantified in SeqMonk using linear read counts. Read counts were exported as ‘Feature Report’ and annotated by gene name. Replicate samples were merged. Transcripts with fewer than 10 informative reads were classified as ‘not expressed’. Transcripts were classified as biallelic when 25%–75% reads originated from the minor allele (i.e., allelic ratio of 3:1).

### Quantification and Statistical Analysis

#### qPCR analysis

Relative quantity was calculated with 2^-ΔΔCt^ using the average value of housekeeping genes *GAPDH* and *RPLPO* (data in Figure S1D) or *GAPDH* and *HMBS* (data in Figure 6C) for ΔCt and the value of primed PSCs for ΔΔCt. Data are presented as mean ± s.d. of 3 or 4 biological replicates. Statistical analysis was done using an ANOVA with Dunnett’s multiple comparison test (GraphPad Prism 7). Significance was accepted with p < 0.05 (^∗^), p < 0.005 (^∗∗^), p < 0.0005 (^∗∗∗^). Statistical details are described in Figure legends.

#### Colony formation assay

In Figure 6D, data are presented as mean ± s.d. of 3 or 4 biological replicates. Statistical analysis was done using an ANOVA with Dunnett’s multiple comparison test (GraphPad Prism 7). Significance was accepted with p < 0.05 (^∗^), p < 0.005 (^∗∗^), p < 0.0005 (^∗∗∗^). Statistical details are described in Figure legends.

#### RNA-sequencing bioinformatics

Differentially expressed genes were identified using DESeq2 with a cut-off of p < 0.05 after multiple testing correction and without independent filtering. For GO analysis, corrected *p-value*s were calculated using a modified Fisher’s exact test followed by Bonferroni’s multiple comparison test. Statistical details are described in Method Details and Figure legends.

### Data and Software Availability

The accession number for the RNA-seq data reported in this paper is GEO: GSE93241.

## Author Contributions

Conceptualization, F.L. and P.J.R.G.; Investigation, S.P.P. and A.J.C. with assistance from A.P.R., J.P.S., and S.P.; Methodology, S.P.P., A.J.C., I.D., and R.W.; Formal Analysis, P.C.; Supervision, F.L., P.J.R.G., and A.E.C.; Writing – Original Draft, A.J.C., S.P.P., F.L., and P.J.R.G.; Writing – Review and Editing, all authors; Funding Acquisition, F.L and P.J.R.G. We consider J.P.S. and P.C. to have contributed equally to this work.

## Supplemental Information

Document S1. Figures S1–S6

Table S1. Summary of Results for the Surface Protein Screen, Related to Figure 1

Table S2. List of Differentially Expressed Genes between Each Cell Type, Related to Figure 7

Table S3. Expression Counts of Transposable Element Classes, Related to Figure 7

Table S4. Details of Antibodies Used for Flow Cytometry and Immunofluorescence Microscopy, Related to Figure 3

Table S5. Information about the Setup of the Flow Cytometers, Related to Figure 3

Table S6. Primer Sequences Used for qRT-PCR, Related to STAR Methods

Document S2. Article plus Supplemental Information
