# Supplementary material for: Comprehensive Cell Surface Protein Profiling Identifies Specific Markers of Human Naive and Primed Pluripotent States
Source: Cell Stem Cell. 2017 Jun 1;20(6):874–890.e7. doi: 10.1016/j.stem.2017.02.014 (PMC5459756; doi:10.1016/j.stem.2017.02.014)
Supplement: Document S1. Figures S1–S6 [file mmc1.pdf]

**Cell Stem Cell, Volume *20***

## Supplemental Information

# Comprehensive Cell Surface Protein Profiling Identifies Specific Markers of Human Naive and Primed Pluripotent States

**Amanda J. Collier, Sarita P. Panula, John Paul Schell, Peter Chovanec, Alvaro Plaza Reyes, Sophie Petropoulos, Anne E. Corcoran, Rachael Walker, Iyadh Douagi, Fredrik Lanner, and Peter J. Rugg-Gunn**

## Supplemental Data

**Comprehensive Cell-Surface Protein Profiling Identifies Novel Markers of Human Naïve and Primed Pluripotent States**

**Collier, Panula et al.**

**Figure S1**, related to Figure 1. Validation of primed and naïve H9 PSCs.

**Figure S2**, related to Figure 1. Experimental set-up for cell-surface marker profiling.

**Figure S3**, related to Figure 2. Validation of individual cell-surface proteins in naïve cells cultured in t2i/L+PKCi and RSeT conditions.

**Figure S4**, related to Figure 3. Characterisation of the cell-surface antibody panel in human PSC lines.

**Figure S5**, related to Figure 6. A multiplexed panel of antibodies to isolate emerging naïve PSCs.

**Figure S6**, related to Figure 6. Transposable elements discriminate between primed, early-stage naïve and established naïve PSCs.

**Table S1**, related to Figure 1. Summary of results for cell-surface protein screen.

**Table S2**, related to Figure 7. List of differentially expressed genes between each cell type.

**Table S3**, related to Figure 7. Expression counts of transposable element classes.

**Table S4**, related to Figure 3. Details of antibodies used for flow cytometry and immunofluorescent microscopy.

**Table S5**, related to Figure 3. Information about the setup of the flow cytometers.

**Table S6,** related to STAR methods. Primer sequences used for RT-qPCR.

Figure S1

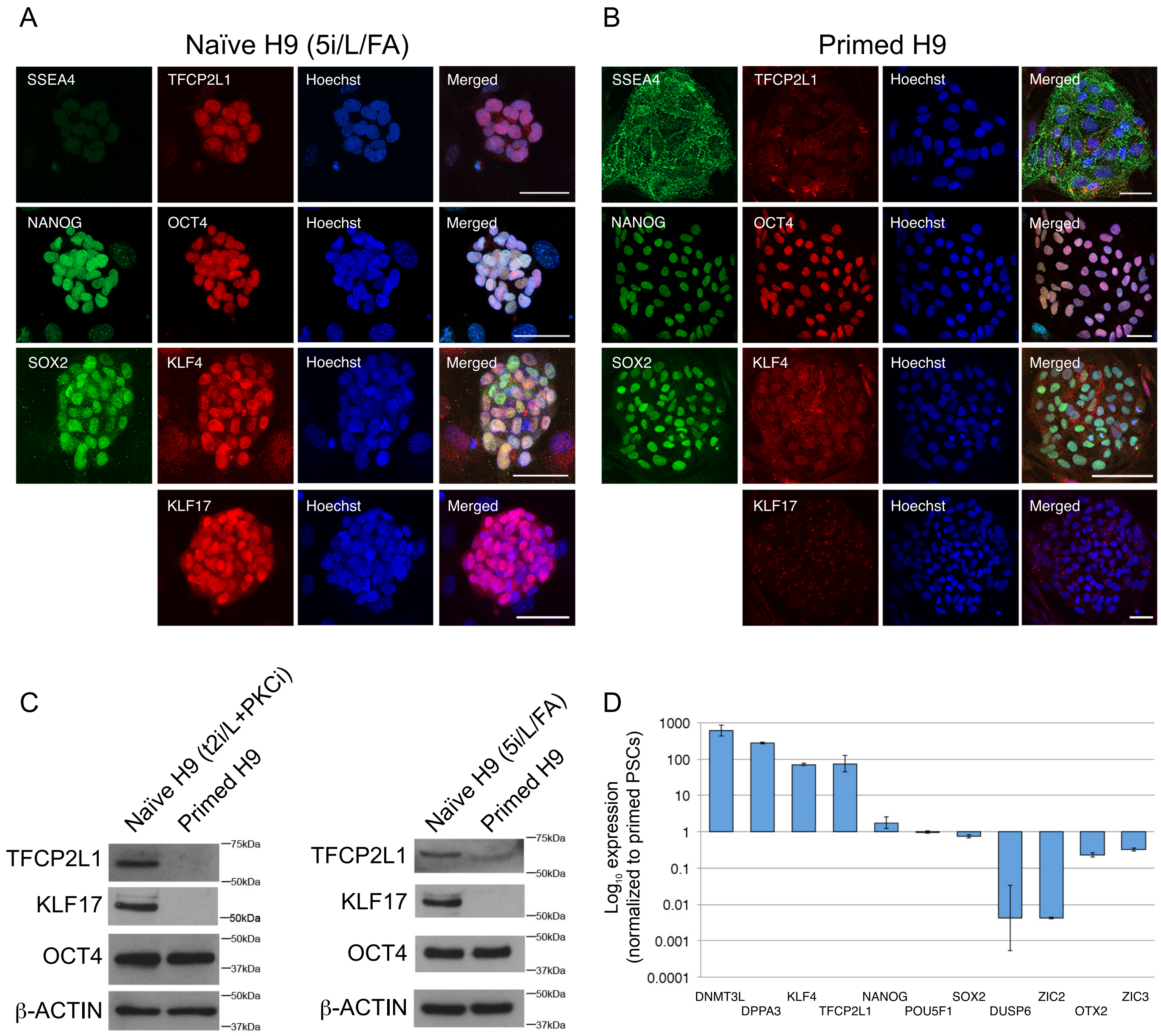

A
Naïve H9 (5i/L/FA)
SSEA4
TFCP2L1
Hoechst
Merged
NANOG
OCT4
SOX2
KLF4
KLF17
B
Primed H9
C
Naïve H9 (t2i/L+PKCi)
Primed H9
Naïve H9 (5i/L/FA)
TFCP2L1
KLF17
OCT4
β-ACTIN
75kDa
50kDa
37kDa
D
Log10 expression (normalized to primed PSCs)
1000
100
10
1
0.1
0.01
0.001
0.0001
DNMT3L
DPPA3
KLF4
TFCP2L1
NANOG
POU5F1
SOX2
DUSP6
ZIC2
OTX2
ZIC3

**Figure S1, related to Figure 1.**

**Validation of primed and naïve H9 PSCs.**

(A-B) Immunofluorescent microscopy of (A) naïve and (B) primed H9 PSCs for pluripotency related proteins. Maximum intensity projections are shown. Scale bars indicate 50 μm.

(C) Western blot analysis of primed and naïve H9 PSCs for the naïve-specific transcription factors TFCP2L1 and KLF17, and a pan-PSC transcription factor OCT4. Left panel shows naïve PSCs cultured in t2i/L+PKCi, and the right panel shows naïve cells cultured in 5i/L/FA. Molecular weight markers are indicated.

(D) Gene expression levels of primed and naïve (5i/L/FA-cultured) H9 PSCs were measured by RT-qPCR for several established naïve and primed PSC genes. Relative expression to housekeeping genes *GAPDH* and *RPLPO*, normalized to primed PSC levels (=1), are shown on $\log_{10}$ scale. Data show mean ± s.d. of 3 biological replicates.

Figure S2

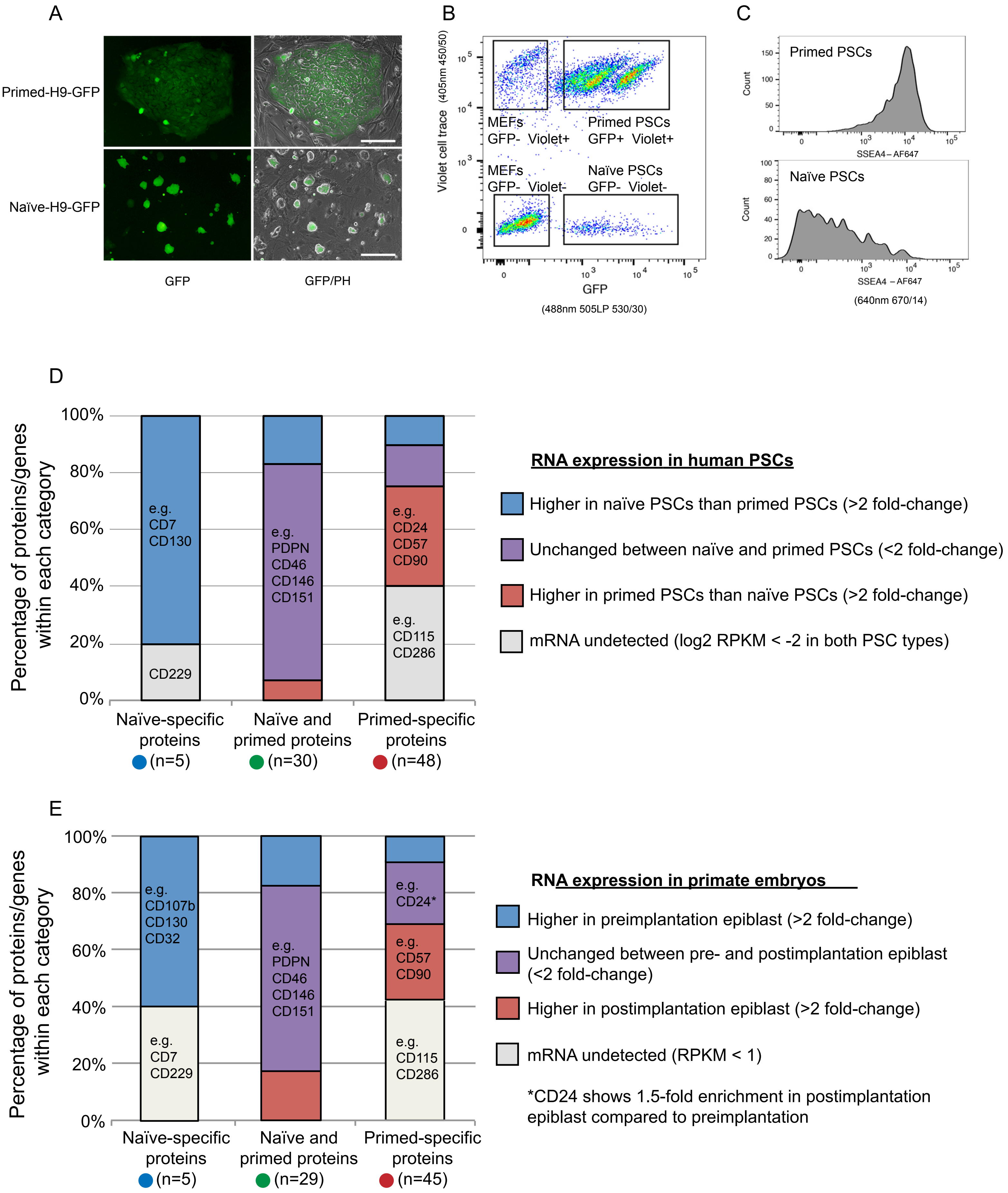
A
Primed-H9-GFP
Naïve-H9-GFP
GFP
GFP/PH
B
Violet cell trace (405nm 450/50)
MEFs GFP- Violet+
Primed PSCs GFP+ Violet+
MEFs GFP- Violet-
Naïve PSCs GFP- Violet-
GFP
(488nm 505LP 530/30)
C
Primed PSCs
Naïve PSCs
Count
SSEA4 – AF647
(640nm 670/14)
D
Percentage of proteins/genes within each category
e.g. CD7 CD130
CD229
e.g. PDPN CD46 CD146 CD151
e.g. CD24 CD57 CD90
e.g. CD115 CD286
Naïve-specific proteins (n=5)
Naïve and primed proteins (n=30)
Primed-specific proteins (n=48)
RNA expression in human PSCs
Higher in naïve PSCs than primed PSCs (>2 fold-change)
Unchanged between naïve and primed PSCs (<2 fold-change)
Higher in primed PSCs than naïve PSCs (>2 fold-change)
mRNA undetected (log2 RPKM < -2 in both PSC types)
E
Percentage of proteins/genes within each category
e.g. CD107b CD130 CD32
e.g. CD7 CD229
e.g. PDPN CD46 CD146 CD151
e.g. CD24*
e.g. CD57 CD90
e.g. CD115 CD286
Naïve-specific proteins (n=5)
Naïve and primed proteins (n=29)
Primed-specific proteins (n=45)
RNA expression in primate embryos
Higher in preimplantation epiblast (>2 fold-change)
Unchanged between pre- and postimplantation epiblast (<2 fold-change)
Higher in postimplantation epiblast (>2 fold-change)
mRNA undetected (RPKM < 1)
*CD24 shows 1.5-fold enrichment in postimplantation epiblast compared to preimplantation

**Figure S2, related to Figure 1.**

**Experimental set-up for cell-surface marker profiling.**

(A) Primed H9 PSCs were transfected with a constitutive GFP expression plasmid and converted to 5i/L/FA naïve PSCs. The GFP signal in the PSCs enables the MEFs (GFP-negative) to be excluded. Representative images are shown for GFP-expressing primed and naïve PSCs. Scale bars indicate 500 μm.

(B) Primed PSCs were labelled with violet cell trace and mixed with unlabelled naïve PSCs prior to immunostaining with the cell-surface marker libraries. This approach enables both cell types to be processed under identical conditions. Flow cytometry plot with gating strategy for different cell populations is shown.

(C) Expression of cell-surface markers was analyzed for primed PSCs ($GFP^+Violet^+$) and for naïve PSCs ($GFP^+Violet^-$) separately. Example of flow cytometry histogram is shown for SSEA4 expression.

(D) Stacked column chart summarises the transcriptional changes of genes that encode for naïve-specific proteins, primed-specific proteins, or proteins expressed by naïve and primed PSCs (defined by the regions shaded in Figure 1B). The number of proteins within each category is shown underneath. Transcript levels were obtained from published RNA-sequencing data (Takashima et al., 2014). Note that not all markers in the flow cytometry screen are encoded by a gene. See Table S1 for protein and transcript values.

(E) Stacked column chart summarises the expression profiles of genes that encode for naïve-specific proteins, primed-specific proteins, or proteins expressed by naïve and primed PSCs (defined by the regions shaded in Figure 1B) in primate pre- and postimplantation embryos. The number of proteins within each category is shown underneath. Transcript levels were obtained from published RNA-sequencing data (Nakamura et al., 2016). Note that not all markers in the flow cytometry screen are encoded by a gene.

Figure S3

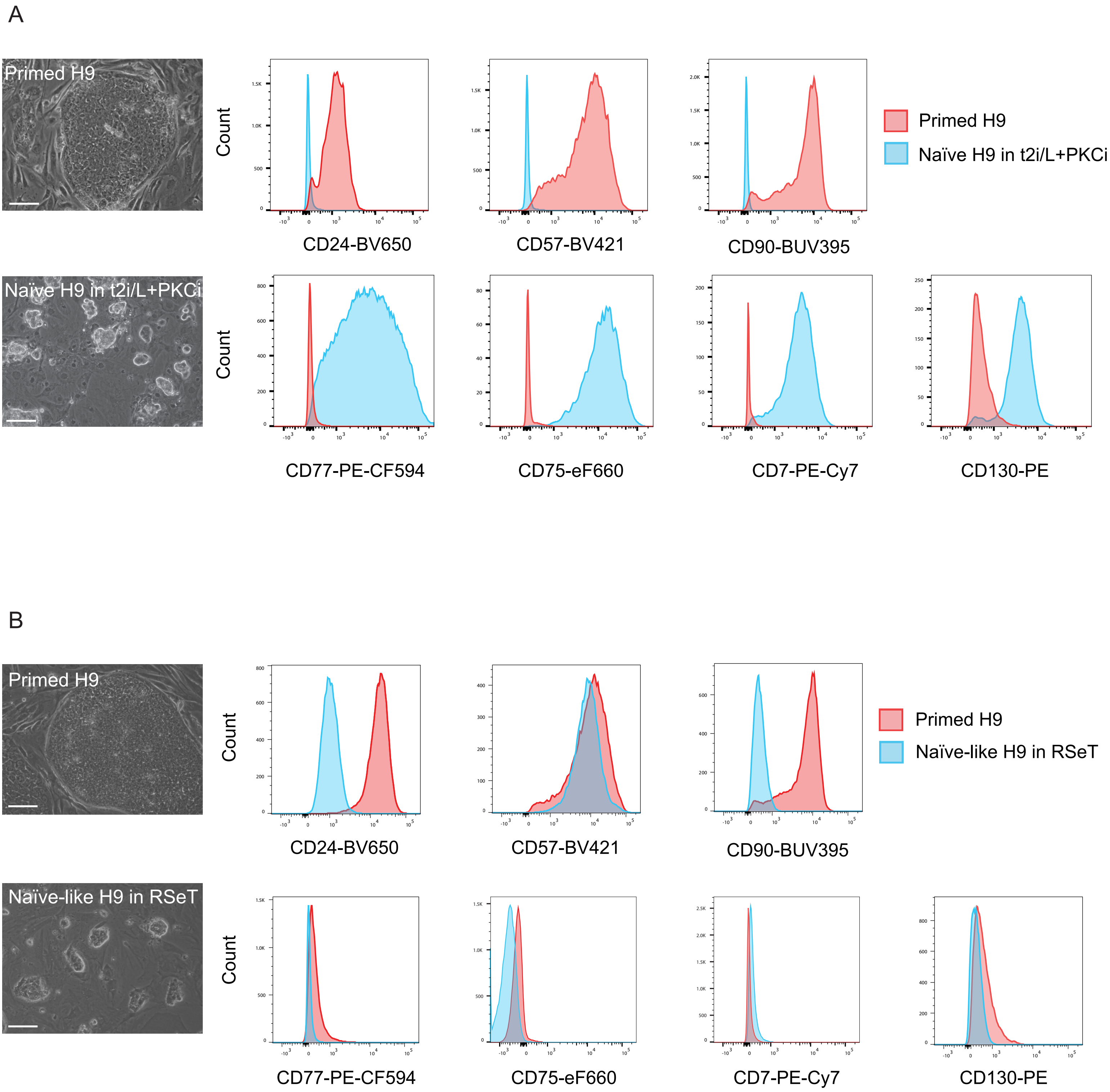
A
Primed H9
Naïve H9 in t2i/L+PKCi
Count
CD24-BV650
CD57-BV421
CD90-BUV395
CD77-PE-CF594
CD75-eF660
CD7-PE-Cy7
CD130-PE
Primed H9
Naïve H9 in t2i/L+PKCi
B
Primed H9
Naïve-like H9 in RSeT
Count
CD24-BV650
CD57-BV421
CD90-BUV395
CD77-PE-CF594
CD75-eF660
CD7-PE-Cy7
CD130-PE
Primed H9
Naïve-like H9 in RSeT

**Figure S3, related to Figure 2.**

**Validation of individual cell-surface proteins in naïve cells cultured in t2i/L+PKCi and RSeT conditions.**

Histograms of flow cytometry analysis using fluorophore-conjugated antibodies show fluorescence signals in H9 primed (red) and naïve (blue) PSCs cultured in (A) t2i/L+PKCi and (B) RSeT conditions. Phase contrast images show representative primed and naïve colonies. Scale bars indicate 100 μm.

Figure S4

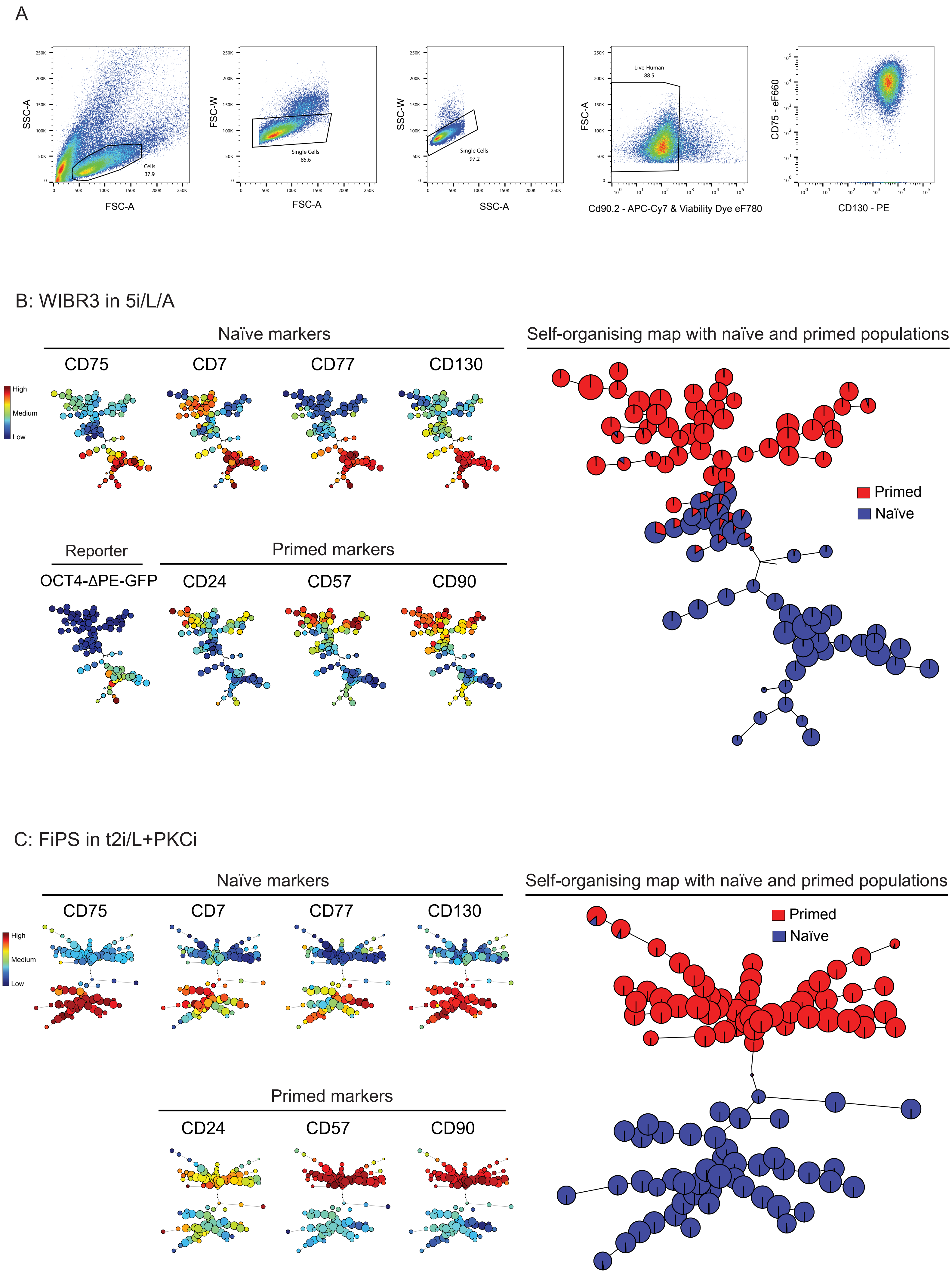
A
SSC-A
FSC-A
Cells
37.9
FSC-W
FSC-A
Single Cells
85.6
SSC-W
SSC-A
Single Cells
97.2
FSC-A
Live-Human
88.5
Cd90.2 - APC-Cy7 & Viability Dye eF780
CD75 - eF660
CD130 - PE
B: WIBR3 in 5i/L/A
Naïve markers
CD75
CD7
CD77
CD130
High
Medium
Low
Reporter
OCT4-ΔPE-GFP
Primed markers
CD24
CD57
CD90
Self-organising map with naïve and primed populations
Primed
Naïve
C: FiPS in t2i/L+PKCi
Naïve markers
CD75
CD7
CD77
CD130
High
Medium
Low
Primed markers
CD24
CD57
CD90
Self-organising map with naïve and primed populations
Primed
Naïve

**Figure S4, related to Figure 3.**

**Characterisation of the cell-surface antibody panel in human PSC lines.**

(A) Flow cytometry dotplots to show gating scheme for H9 naïve PSCs. The first panel enables the discrimination of cells versus debris, and the next two panels identify single cells. In the fourth panel, to select for live, human cells a gate was placed to exclude Cd90.2-positive mouse feeder cells and dead cells using an eF780 Viability Dye. The last panel provides an example to show that the final gated population are positive for naïve-state markers CD75 and CD130.

(B–C) FlowSOM visualisation of flow cytometry data for (A) WIBR3 PSCs cultured in 5i/L/A and (B) FiPS PSCs in t2i/L+PKCi. An unsupervised self-organizing map arranges the cells into clusters (represented by circles) according to similarities in their cell-surface protein expression profiles (right panels). Overlaying the name of the cell-type within each cluster reveals a clear separation of naïve (blue) and primed (red) populations. The heatmap panels show the expression level of each cell-surface protein in the cell clusters (left). Clusters are arranged in the same position as for the minimal spanning tree of the self-organizing map.

Figure S5

Primed-state to naïve-state conversion with FACS populations (day 10)

A

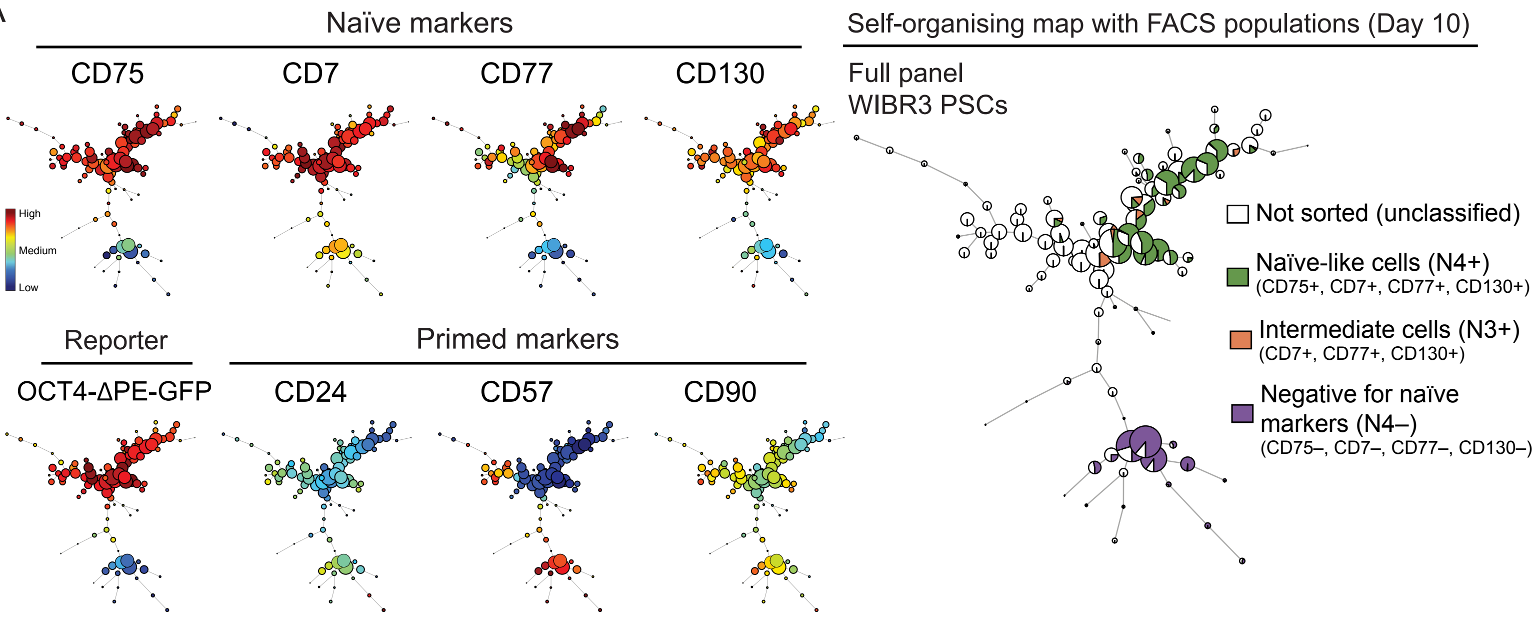


B

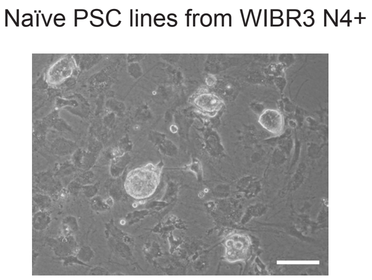


C

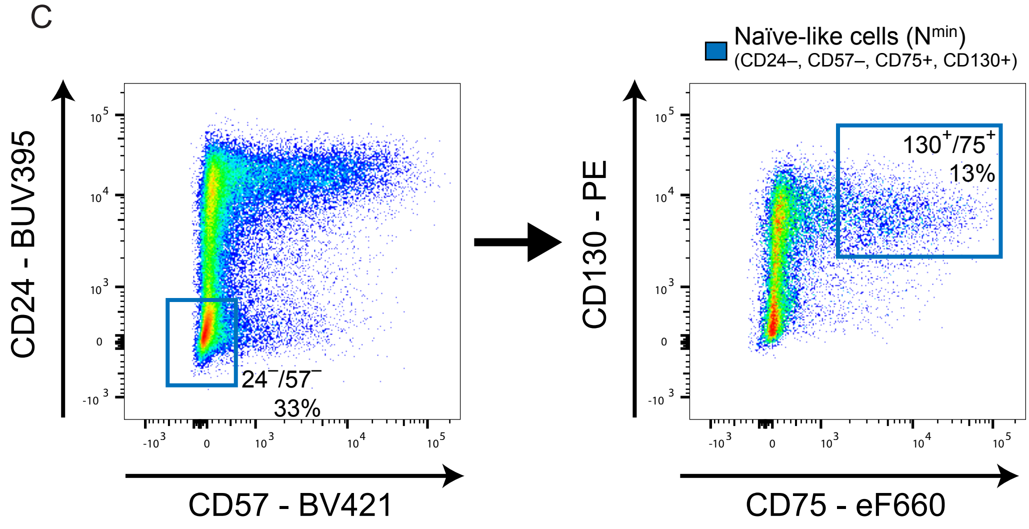


D

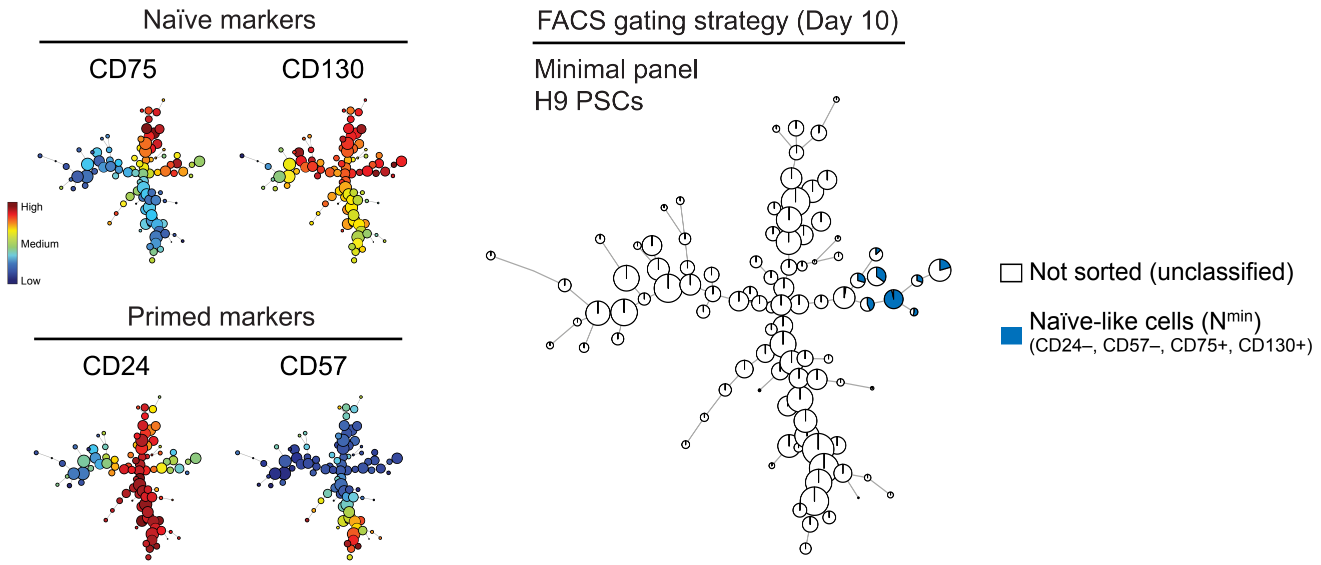

**Figure S5, related to Figure 6**

**A multiplexed panel of antibodies to isolate emerging naïve PSCs.**

(A) FlowSOM visualisation of the flow cytometry data for day 10 cells during primed-state to naïve-state conversion of WIBR3 PSCs using 5i/L/A-mediated resetting. The minimal spanning tree of the self-organizing map displays an unsupervised clustering of the sample based on the cell-surface protein expression levels (right panel). The cells corresponding to each cell sorting population, N4+, N3+ and N4–, are indicated. The heatmap panels show the expression level of each cell-surface protein marker in the cell clusters (left).

(B) Phase contrast image shows a representative field of view of WIBR3 N4+ cell sorted population that have been propagated in 5i/L/FA naïve PSC conditions for three passages. Scale bar indicates 100μm.

(C) A minimal antibody panel to isolate emerging naïve PSCs. Flow cytometry dotplots of day 10 cells during primed-state to naïve-state conversion of H9 PSCs in t2i/L+PKCi conditions. The left panel shows the levels of two primed-specific proteins CD24 and CD57. A cell sorting gate has been drawn that corresponds to CD24–/CD57– (blue box) cell populations. The right panel shows the levels of the naïve-specific proteins CD130 and CD75 proteins for the same gated cell population. The boxed area indicates the $N^{min}$ (blue) cell population that was used for subsequent experiments. In both panels, the percentage of cells within each cell sorting gate relative to all live, human cells is shown.

(D) FlowSOM visualisation of the flow cytometry data for day 10 cells during primed-state to naïve-state conversion. H9 cells were interrogated using a minimal panel of antibodies that target two naïve-specific proteins (CD75 and CD130) and two primed-specific proteins (CD24 and CD57). The minimal spanning tree of the self-organizing map displays an unsupervised clustering of the sample based on the cell-surface protein expression levels (right panel). The

cells corresponding to the $N^{min}$ cell sorting population is indicated. The heatmap panels show the expression level of each cell-surface protein marker in the cell clusters (left).

Figure S6

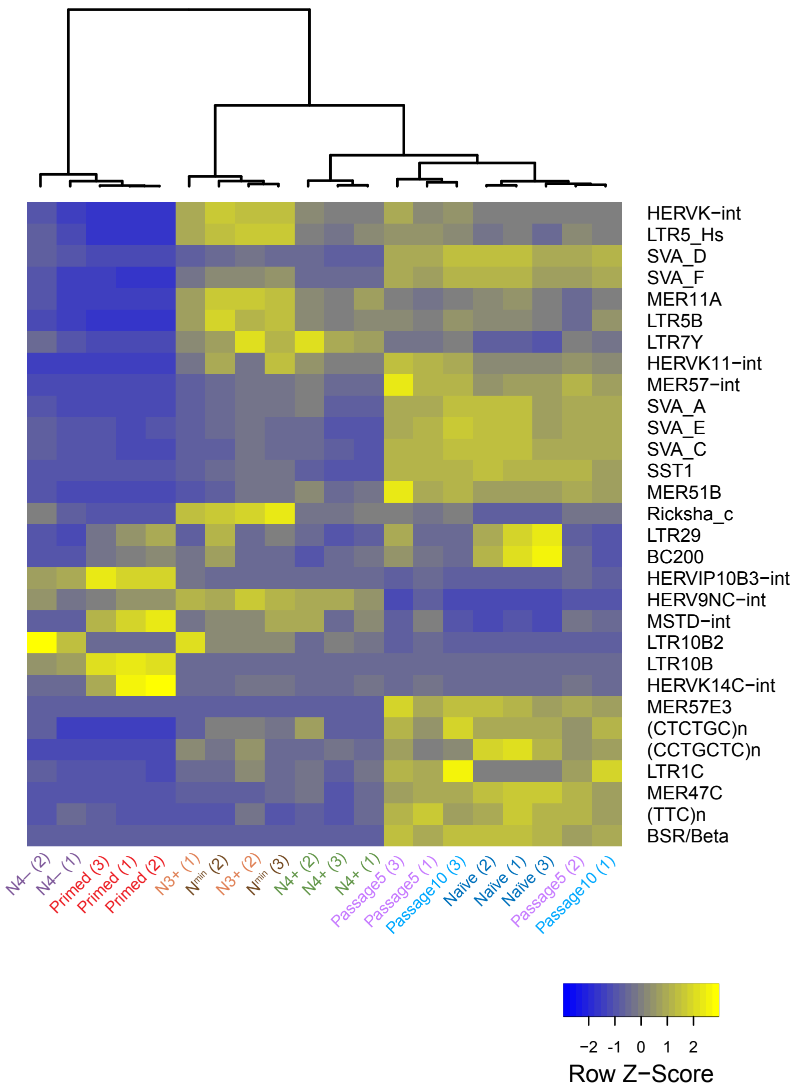

HERVK−int
LTR5_Hs
SVA_D
SVA_F
MER11A
LTR5B
LTR7Y
HERVK11−int
MER57−int
SVA_A
SVA_E
SVA_C
SST1
MER51B
Ricksha_c
LTR29
BC200
HERVIP10B3−int
HERV9NC−int
MSTD−int
LTR10B2
LTR10B
HERVK14C−int
MER57E3
(CTCTGC)n
(CCTGCTC)n
LTR1C
MER47C
(TTC)n
BSR/Beta
N4− (2)
N4− (1)
Primed (3)
Primed (1)
Primed (2)
N3+ (1)
N$^{min}$ (2)
N3+ (2)
N$^{min}$ (3)
N4+ (2)
N4+ (3)
N4+ (1)
Passage5 (3)
Passage5 (1)
Passage10 (3)
Naïve (2)
Naïve (1)
Naïve (3)
Passage5 (2)
Passage10 (1)
−2 -1 0 1 2
Row Z−Score

**Figure S6, related to Figure 7**

**Transposable elements discriminate between primed, early-stage naïve and established naïve PSCs.**

Hierarchical clustering of transposable element expression data. Samples (columns) and transposable element classes (rows) were clustered based on Euclidean distance. The top 30 most variable transposable elements across all samples are shown. The expression data (RPM normalised) are presented as Z-score values varying from yellow (high) to blue (low). See Table S3 for transposable element expression data set.

**Table S1, related to Figure 1. Summary of results for cell-surface protein screen.** Results shown are the average percent positive values for naïve-state and primed-state H9 PSCs. Also shown are transcript levels of genes that encode for naïve-specific proteins, primed-specific proteins, or proteins expressed by naïve and primed PSCs. Transcript levels are shown as log2 RPKM (Takashima et al., 2014).

**Table S2, related to Figure 7. List of differentially expressed genes between each cell type compared to established naïve PSCs, and of differentially expressed genes between N3+ and N4+ samples.** Transcript levels are shown as log2 RPKM

**Table S3, related to Figure 7. Expression counts of transposable element classes (RPM).**

**Table S4, related to Figure 3. Details of antibodies used for flow cytometry and immunofluorescent microscopy.** Antibodies multiplexed in the full and minimal panels are indicated.

**Table S5, related to Figure 3. Information about the setup of the flow cytometers including lasers, filters and fluorochrome details.**

**Table S6, related to STAR methods. Primer sequences used for RT-qPCR.**
